# Highly Sensitive Water Detection Through Reversible Fluorescence Changes in a *syn*-Bimane Based Boronic Acid Derivative

**DOI:** 10.3389/fchem.2021.782481

**Published:** 2022-01-17

**Authors:** Apurba Pramanik, Joy Karmakar, Flavio Grynszpan, Mindy Levine

**Affiliations:** Department of Chemical Sciences, Ariel University, Ariel, Israel

**Keywords:** bimane, fluorescence, water sensor, boronate ester, paper-based sensing

## Abstract

Reported herein is a fluorometric and colorimetric sensor for the presence of trace amounts of water in organic solvents, using *syn*-bimane based boronate ester **1**. This sensor responds to the presence of water with a highly sensitive turn-off fluorescence response, with detection limits as low as 0.018% water (v/v). Moreover, analogously high performance was observed when compound **1** was adsorbed on filter paper, with the paper-based sensor responding both to the presence of liquid water and to humid atmospheres. Reusability of the paper-based sensor up to 11 cycles was demonstrated, albeit with progressive decreases in the performance, and ^1^H NMR and mass spectrometry analyses were used to explain the observed, hydrolysis-based sensor response.

**GRAPHICAL ABSTRACT:**
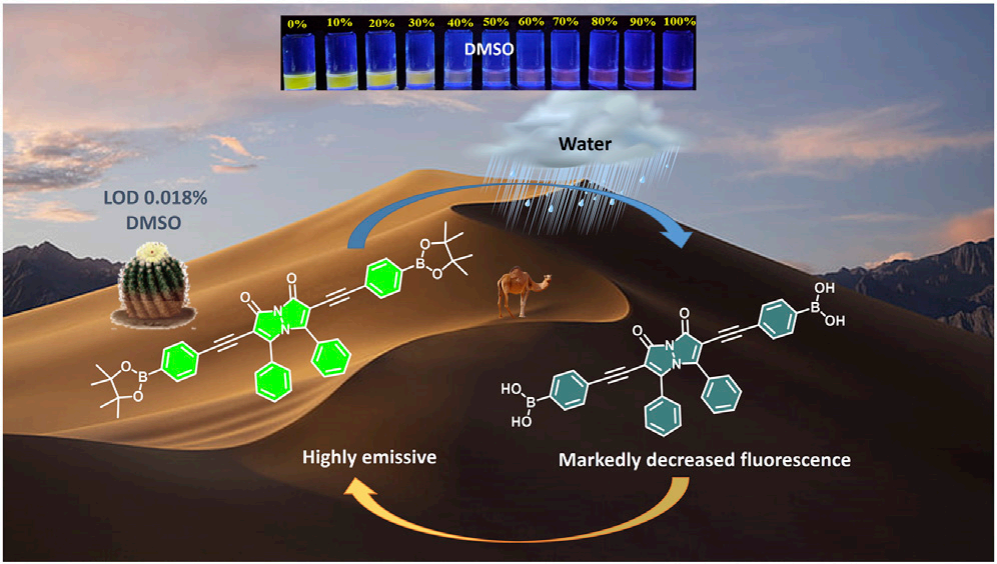


## Introduction

The detection of water in organic solvents is of significant interest from a variety of industrial, pharmaceutical, and chemical safety perspectives (Mishra and Singh 2021). From an industrial perspective, even minute quantities of water in organic solvents can rapidly contaminate chemical reactions, leading to deactivation of catalysts, a reduction in reaction efficiencies, and the generation of undesired side products (Lin et al., 2012). From a pharmaceutical perspective, the presence of water in organic solvents can dramatically affect the optimized syntheses of pharmaceutically active agents, with significant financial consequences particularly for large-scale syntheses (Thenrajan et al., 2021). From a chemical safety perspective, the incompatibility of a variety of molecules with water is well-known (Zor et al., 2021), and inadvertent exposure to water found as a contaminant in organic solvents can lead to the violent decomposition of water-incompatible reagents, resulting in chemical explosions and/or other undesired highly exothermic reactions (Yang et al., 2019).

Current methods for the detection of water in organic solvents tend to rely on spectral monitoring of the solvent to detect signals that correspond to the presence of water, and include the use of FTIR (Saleh and Tripp 2021), Raman (Yeung and Chan 2010), UV-visible (Mohar 2019), and fluorescence spectroscopy (Zhou et al., 2019; Kumar et al., 2016). Other methods, including gas chromatography (Kay et al., 2021) and electrochemical methods (Marecek and Samec 2020), have also been reported. These methods, which rely either on the detection of water directly or on the detection of signal changes corresponding to other molecules that interact with water, generally lead to extremely high sensitivity and selectivity (Wang et al., 2021). Nonetheless, ongoing challenges in using such methods include the need for costly and non-portable laboratory-grade instruments to monitor such spectral changes, as well as the fact that many of these methods are not reversible. Moreover, toxicity concerns remain around the use of water-detection materials that contain heavy metals, such as mercury and cadmium (Othong et al., 2020); around the use and responsible disposal of sensors composed of graphene oxide (Chi et al., 2021), carbon dots (Lee et al., 2019), and other carbon-based materials (Liu et al., 2020); and around sensors that rely on relatively weak noncovalent interactions in the sensor construction (Wu et al., 2020), and the potential for the degradation of such sensors to lead to environmental contamination (Gao et al., 2010).

Recent reports from one of our research groups have demonstrated the development of high-performance fluorometric (Haynes and Levine 2020) and colorimetric (Haynes et al., 2019) sensors for a variety of organic and inorganic analytes. Moreover, recent successes in novel sensor development have been reported in joint publications from our research groups, and demonstrate that bimane-based supramolecular constructs act as highly effective sensors for cobalt (II) ions (Pramanik et al., 2020) and for molecular iodine (Pramanik et al., 2021). These sensors, which operate in both solution-state and on filter papers to provide fluorometric and colorimetric analyte detection, have notable practical advantages, including their high sensitivity, ease of access, and non-toxicity of both the bimane transducing element and supramolecular cyclodextrin scaffold. The lack of toxicity of both cyclodextrin (Kfoury et al., 2019) and bimane (MSDS 38369) has been well-established in the literature, and has led to the use of both of these components in a variety of biologically-relevant applications (Barbosa et al., 2019; Lapidot et al., 2016; Lavis and Raines 2008). Moreover, an additional report from one of our research groups has demonstrated a streamlined approach to access bimane derivatives, which facilitates practically attainable access to a variety of these highly fluorescent structures (Szumski et al., 2021). This straightforward access, combined with our already demonstrated ability to use bimanes as components of effective sensors (Roy et al., 2018), prompted us to investigate the use of bimane derivatives as high impact fluorescent sensors for water contamination. Moreover, a recent theoretical report indicated that bimane is expected to demonstrate significant water-induced fluorescence quenching, although experimental evidence in support of these calculations was not provided (Maillard et al., 2021).

Reported herein are the results of our investigations, which demonstrate that bimane **1** responds to the presence of liquid water in organic solvents and water vapor in high humidity environments, and that such a response occurs both in solution of bimane **1** and on filter papers to which bimane **1** had been adsorbed, leading to colorimetric and fluorimetric changes. Although water sensing via a similar boronate hydrolysis mechanism has been demonstrated using other fluorophores (Selvaraj et al., 2019), it has not yet been demonstrated using the bimane scaffold and represents a significant step towards the establishment of bimanes as a viable option in the tool-box of available fluorophores (Kosower et al., 1979). Determining the linear relationship between the sensor response (either colorimetric or fluorometric) and the amount of water provides a method for its straightforward quantitation. Finally, good reversibility of the paper-based sensors was also demonstrated, as were detailed mechanistic investigations that explain the basis of the sensor response.

## Materials and Methods

### Synthesis of Bimane 1

Compound **1** was synthesized from compounds **3** and **4**. Bis(triphenylphosphine) palladium (II) chloride (20 mg, 0.028 mmol, 0.10 equiv) and cuprous iodide (2.7 mg, 0.014 mmol, 0.05 equiv) were added to a solution of 4-ethynylphenylboronic acid pinacol ester **4** (140 mg, 0.61 mmol, 2.2 equiv), diisopropylethylamine (0.48 ml, 2.8 mmol, 10 equiv), and compound **3** (150 mg, 0.28 mmol, 1.0 equiv) in CH_3_CN (200 ml). The mixture was stirred at 80 °C for 1 hour under a nitrogen atmosphere. After 1 hour, the solvent was evaporated under reduced pressure, and the resulting crude product was purified *via* flash chromatography over silica gel eluting with 5% ethyl acetate: 95% dichloromethane. The product was isolated as a reddish-yellow colored solid in 67% yield (138 mg). ^1^H NMR (CDCl_3_): 7.72−7.70 (*d*, *J* = 8 Hz, 2H, Ar-H), 7.36−7.34 (*d*, *J* = 8 Hz, 2H, Ar-H), 7.32-7.28 (m, 2H, Ar-H), 7.24-7.23 (*d*, *J* = 4 Hz, 1H, Ar-H), 7.17−7.13 (*m*, 2H, Ar-H), 1.33 [*s*, 12 H, 2(-Me)_2_] ppm; ^13^C NMR (CDCl_3_): 134.64, 131.24, 130.89, 129.31, 128.29, 84.15, 25.01 ppm; DEPTQ: 134.96, 131.20, 129.63, 128.61, 126.12, 25.32 ppm; HRMS *m/z* [M + H]^+^ calculated: 741.3322, found: 741.3332.

### UV-Visible Spectroscopy Procedures

UV-Visible absorption spectroscopy was used in two different situations: 1) to study the solvent dependent properties of the bimane-based boronate compound **1**; and 2) to investigate the detection of water using bimane-based boronate compound **1**. Procedures for each of these situations are detailed below.

#### To Study the Solvent Dependent Properties of Compound 1

The effects of 13 different solvent systems were investigated by measuring the UV-visible absorption spectra of bimane **1** (from 200 to 700 nm) at a concentration of 10 µM in each of the solvent systems (see Supplementary Table S1).

#### To Investigate the Detection of Water Using Compound 1

The changes of the UV-visible absorbance spectrum of compound **1** upon the addition of varying concentrations of water was measured in a variety of water-miscible solvents. These experiments were conducted in HPLC grade solvents, with the concentration of compound **1** held constant at 10 μM, and with increasing concentrations of Milli-Q purified water added to the solution.

### Fluorescence Spectroscopy Procedures

Fluorescence spectroscopy was used to investigate two different situations: 1) to study the solvent dependent fluorescence properties of compound **1**; and 2) to investigate the detection of water using compound **1** in a variety of solvents. In all cases, the excitation of bimane **1** occurred at 450 nm, and the excitation and emission slit widths were both 5.0 nm. The procedures used in each of these situations are discussed in detail below:

#### To Study the Solvent Dependent Fluorescence Properties of Compound 1

The concentration of compound **1** was held constant at 10 µM in acetonitrile, acetonitrile-water (1:1 vol: vol), methanol, ethanol, tetrahydrofuran, ethyl acetate, dichloromethane, chloroform, acetone, diethyl ether, *N,N*-dimethylformamide (DMF), dimethylsulfoxide (DMSO), and water. The fluorescence emission of compound **1** in each solvent was recorded via excitation at 450 nm. Changes in the fluorescence emission of bimane **1** were quantified by integrating the fluorescence emission vs. wavenumber on the X-axis (using OriginPro 2020).

#### To Investigate the Detection of Water Using Compound 1

Increasing concentrations of water were added to solutions of compound **1** in organic, water-miscible solvents, with the concentration of bimane **1** held constant at 10 µM. The concentration of water in these experiments ranged from 0 to 43.16 µM. In all cases, changes in the fluorescence emission of compound **1** under these conditions were quantified by integrating the fluorescence emission vs. wavenumber on the X-axis using OriginPro 2020.

### Experimental Procedures for Paper-Based Studies

Whatman #1 filter papers with dimensions of 3.5 cm × 0.9 cm were coated with a solution of compound **1** by submerging the filter papers in an acetonitrile solution of compound **1** [(**1**) = 10 µM] for 60 min at room temperature. After 60 min, the papers were carefully removed from the solution using tweezers and were then placed on a Petri dish and allowed to dry for 3 hours in an open-air environment. After that, varying amounts of water (100, 150, 200, 250, 300, or 350 µL) were added via pipette to the top of the paper, and the paper was allowed to dry for 4 h at room temperature on a benchtop. The dried papers were then visualized under a long-wave, hand-held TLC lamp (365 nm excitation) and the results of these studies are reported herein.

## Results and Discussion

**FIGURE 1:**
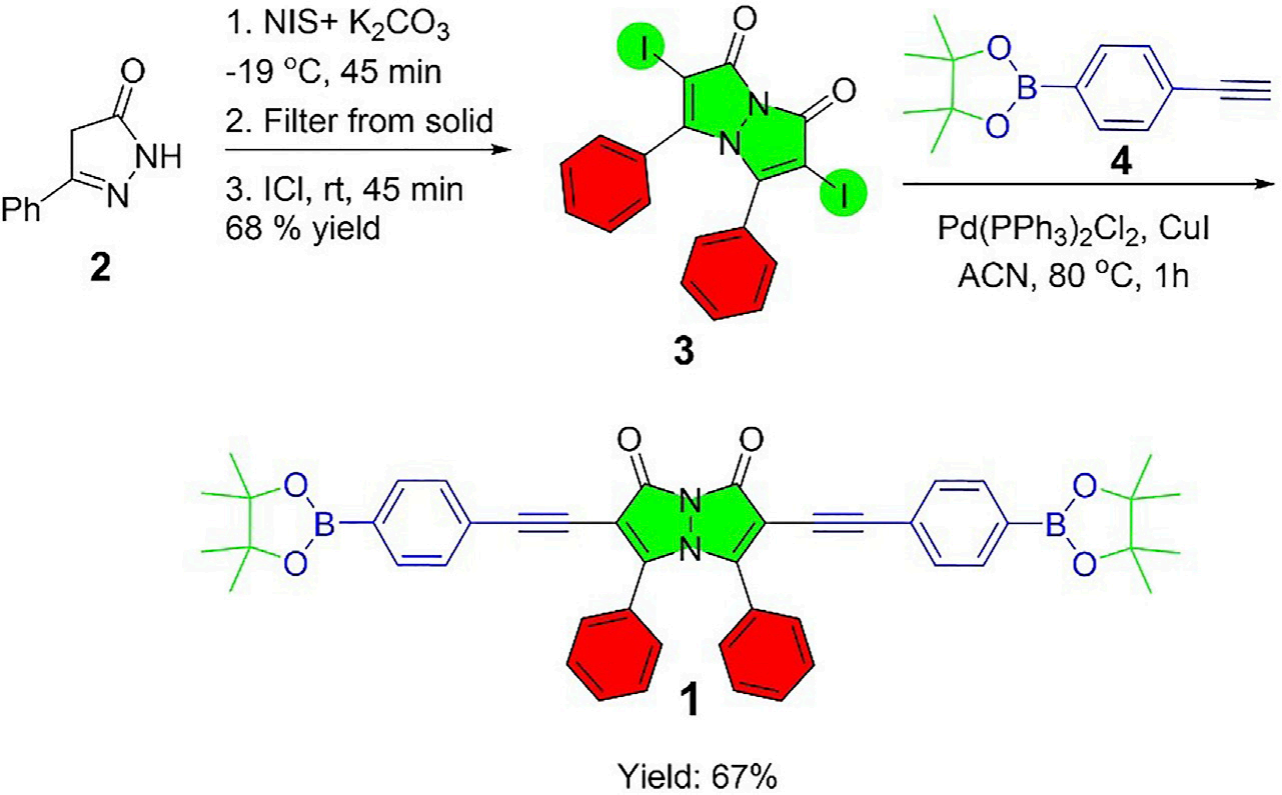
A streamlined synthesis of diboronate ester functionalized bimane **1**.

The fluorescent sensor for water reported herein relies on bimane **1**, which was synthesized via the Sonogashira coupling of diiodobimane **3** with two equivalents of acetylene **4** (Figure 1). This diidobimane was in turn accessed from 5-phenyl-4,4-dihydropyrazol-3-one **2** in a one-pot, three-step sequence in 68% overall yield, representing a marked improvement over multi-step, literature-reported methods that require the use of toxic chlorine gas and provide low overall yields (Kosower et al., 1982). The relative quantum yield of bimane **1** (0.17) and its extinction coefficient (1.23 × 10^4^ L M^−1^ cm^−1^) are somewhat lower than values reported for other bimane structures (i.e., 0.83 for an unsymmetrically substituted bimane containing one chlorine substituent) (Szumski et al., 2021), but are in line with Kosower’s observation that electron-donating alkyl and aryl substituents on the *syn*-bimane structure lead to noticeable reductions in the quantum yield (Kosower et al., 1983).

**FIGURE 2:**
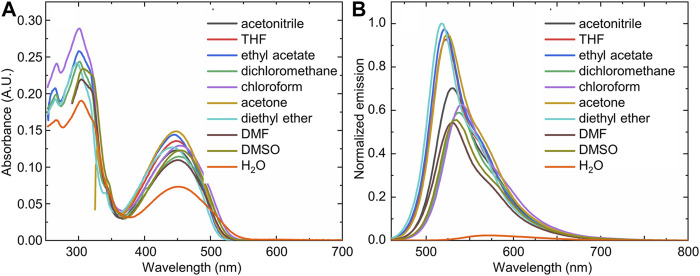
The **(A)** UV-visible and **(B)** fluorescence emission spectra of compound **1** in a variety of solvents, highlighting the marked difference in spectra observed in water (orange trace) compared to the organic solvents [(**1**) = 10 μM; *λ*_ex_ = 450 nm].

Interestingly, compound **1** displayed mild solvatochromism in its steady-state photophysical measurements, with slight solvent-induced differences in the absorbance (Figure 2A) and emission (Figure 2B) spectra observed. These changes were markedly more pronounced in a fully aqueous solvent, with water leading to a strong decrease in the visible absorbance band of compound **1**, and an even more pronounced decrease in the fluorescence emission. Interestingly, compound **1** in the solid-state also displayed a strong fluorescence emission, which decreased markedly upon the addition of water.

**FIGURE 3:**
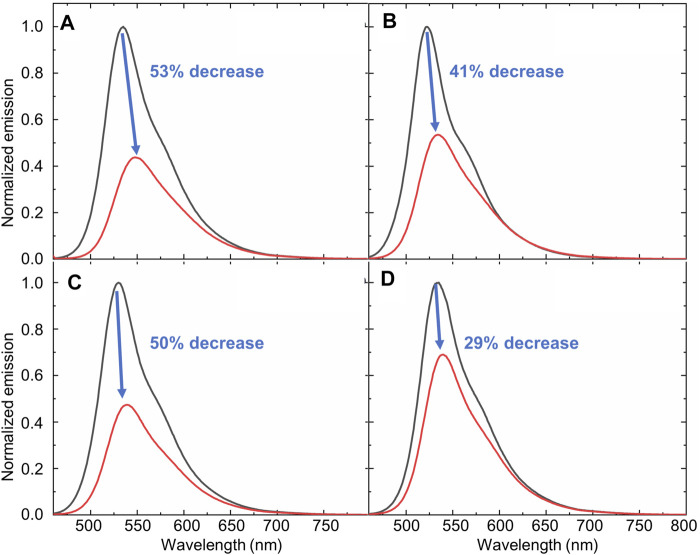
An illustration of the changes in the fluorescence emission of compound **1** that occurs in the absence (black line) or presence (red line) of water in water-miscible organic solvents: **(A)** Acetonitrile; **(B)** THF **(C)** DMF; and **(D)** DMSO. [(**1**) = 10 μM; *λ*_ex_ = 450 nm].

This water-induced quenching displayed a strong solvent-dependent response (Figure 3), with the addition of water to acetonitrile displaying the greatest degree of water-induced fluorescence quenching (Figure 3A, 53% decrease in the integrated fluorescence emission compared to the emission in the absence of water). Extremely high sensitivity for low concentrations of water was also demonstrated (Table 1), with the lowest detection limit for water of 0.018% (v/v) calculated in DMSO. This detection limit is in line with the limits of the most sensitive sensors for water in organic solvents reported to date (Huang et al., 2021), and indicates that the sensor reported herein represents a highly sensitive detection method (Kumar et al., 2021). Of note, all aqueous studies were conducted in double deionized water, so that no interference and/or fluorescence quenching occurred due to residual metal ions. Such ions, in particular palladium (Das et al., 2016), sodium (Roy et al., 2018), and cobalt (Pramanik et al., 2020), have been shown to complex effectively to the bimane core, leading to marked changes in the bimane’s photophysical properties. Also of note, the existence of trace amounts of water even in the “0%” water is likely, based on literature precedent (Son et al., 2001), but this trace amount of water is accounted for as part of the baseline for our fluorescence quenching experiments, in accordance with literature precedent (Porter and Markham 1970).

**TABLE 1:** Limits of detection and quantification of water in organic, water-miscible solvents, calculated as the percentage of water by volume.^a^

Solvent	Limit of detection (v/v)	Limit of quantification (v/v)
Acetonitrile	0.57 ± 0.003%	0.96 ± 0.007%
THF	5.74 ± 0.014%	6.05 ± 0.014%
DMF	0.17 ± 0.007%	0.60 ± 0.014%
**DMSO**	**0.018 ± 0.001%**	**0.19 ± 0.007%**
Acetone	0.76 ± 0.001%	1.08 ± 0.002%

a Limits of detection and quantification were calculated as percentage of water by volume in the solvent system, and results reported herein represent the average of at least three trials. The text in bold highlights the solvent with lowest LOD

**FIGURE 4:**
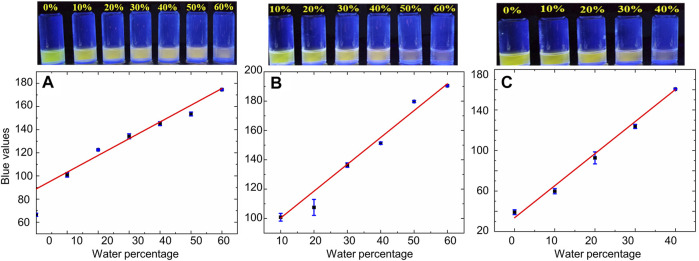
Top: Naked eye color changes induced by the addition of increasing percentages of water to water-miscible solvents. Bottom: The linear relationship between the added water percentage and the quantitative blue value of the solution, measured from photographs taken under 365 nm excitation. **(A)** Acetonitrile solutions; **(B)** DMF solutions; and **(C)** DMSO solutions (other solvents are shown in the ESI; blue bars represent the calculated standard deviations for each data point).

Moreover, this highly sensitive fluorescence-based quenching response was accompanied by noticeable changes in the color of bimane **1** solutions in varying solvents with increasing percentages of water, which were particularly visible using 365 nm excitation of the bimane **1** solutions. Examples of such dramatic color changes are shown for water added to acetonitrile, THF, and DMSO (Figure 4, top). The quantitative blue values changed as a result of the addition of increasing percentages of water in a manner that was linear for a broad range of water percentages (Figure 4, bottom, Table 2); such linearity, in turn, directly enables the ability to detect unknown water concentrations within such solvent systems. Finally, general applicability for the system was also demonstrated, with both purified milli-Q water and aqueous buffer solutions between pH 5 and pH 7.5 inducing identical colorimetric responses (see ESI for more details).

**TABLE 2:** The linear relationship between the percentage of water added to solutions of bimane **1** in water-miscible solvents and the quantitative blue value of the resulting solution when imaged under 365 nm excitation.^a^

Solvent	Linear range	Equation	*R* ^2^ value
Acetonitrile	0–60% water	y = 15.864x + 64.5	0.9509
THF	0–60% water	y = 115.75x + 71.518	0.9207
DMF	10–60% water	y = 198.15x + 73.953	0.9841
DMSO	0–40% water	y = 313.7x + 30.98	0.9897
Acetone	0–100% water^b^	y = -190.15x + 221.16	0.9249

a Data were obtained from photographs of the bimane **1** solution under long-wave TLC, light irradiation (365 nm), with processing using random sampling of data points with Microsoft Paint (>10 random points/sample) and linear curve fitting using Microsoft Excel; (**1**) = 10 µM.

b Green values were used as the blue values gave a poor linear fit.

**FIGURE 5:**
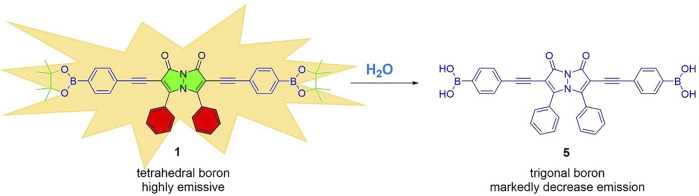
The hydrolysis of boronate ester **1** to boronic acid **5**, which likely is responsible for water-induced fluorescence decreases.

The mechanism by which the addition of water induces the fluorescence quenching of bimane **1** is likely via the water-induced hydrolysis of the boronate ester moieties on compound **1** to form the hydrolyzed boronic acid **5** (Figure 5). The formation of compound **5** was confirmed by ^1^H NMR titrations and high-resolution mass spectrometry (see ESI for more details). Moreover, an analogous hydrolysis reaction occurred in methanol, resulting in the formation of methyl ester-substituted boronate moieties in lieu of the pinacol boronates. The fact that changing the hybridization of boron-containing moieties from tetrahedral to trigonal planar induces such marked fluorescence decreases has been previously reported in the literature (Samaniego Lopez et al., 2015; Williams et al., 2021), and is fully consistent with the results reported herein. Notably, this fluorescence quenching occurs rapidly (within seconds), and remains stable (with less than 5% additional quenching) up to 20 min after the initial contact between compound **1** and water.

**FIGURE 6:**
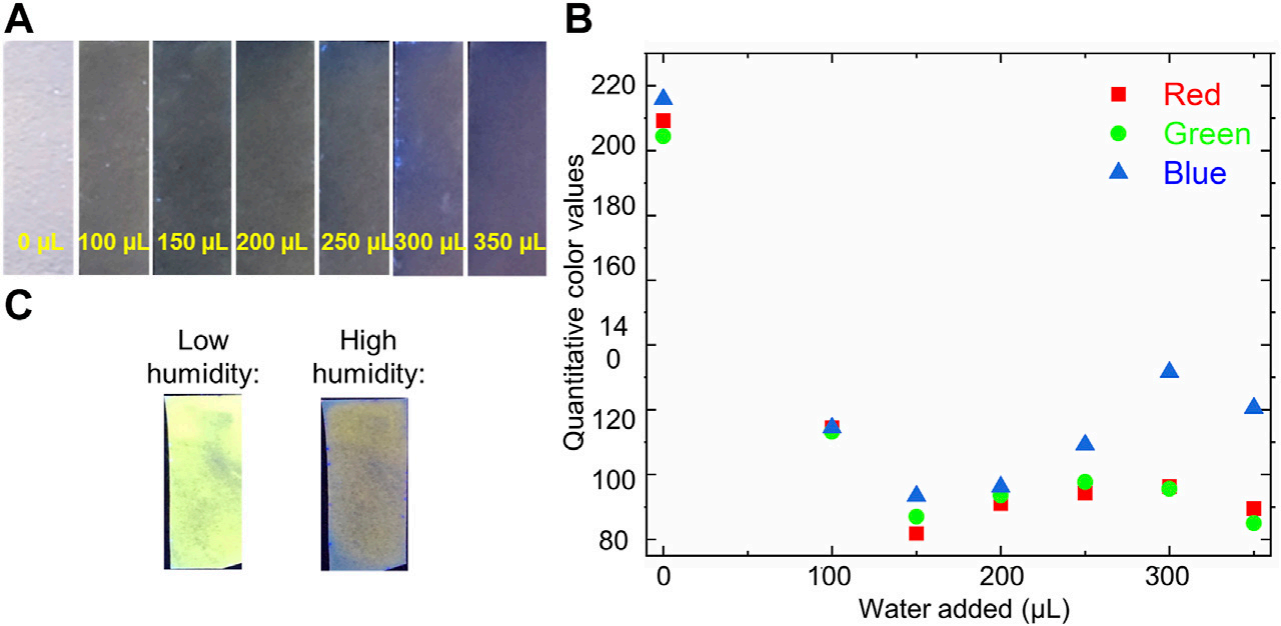
**(A)** Photograph of bimane **1**-functionalized filter paper after the addition of water via micropipette (left-to-right: 0, 100, 150, 200, 250, 300, and 350 µL) **(B)** Quantitative RGB values of the bimane **1**-functionalized filter paper as a function of the addition of increasing amounts of water (calculated via ImageJ analysis); **(C)** Photographs of bimane **1**-functionalized filter paper under low humidity and high humidity conditions, imaged under 365 nm excitation.

Gratifyingly, moving from solution-state water sensing to sensing on a solid-support resulted in analogous water-induced photophysical changes, with the addition of water to filter paper onto which bimane **1** had been adsorbed causing a marked change in the color and fluorescence of the system (Figure 6A). Notably, the procedure by which water is added to these paper sensors (dropping *via* pipette onto the functionalized paper) is done by design to ensure that we control the amount of water that the paper contacts, but we recognize that such a procedure will require further optimization before real-world device development and deployment. These changes were quantified by RGB color analysis (Figure 6B), which shows that the system becomes rapidly saturated with water, resulting in minimal changes in the RGB values after 100 µL of water were added. Even more significantly, bimane **1**-functionalized filter papers responded to the presence of water vapor with an analogously strong sensor response, with exposure of the functionalized paper to a high humidity chamber (99% relative humidity) resulting in analogous changes in the sensor’s photophysical (colorimetric and fluorometric) profile (Figure 6C).

**FIGURE 7:**
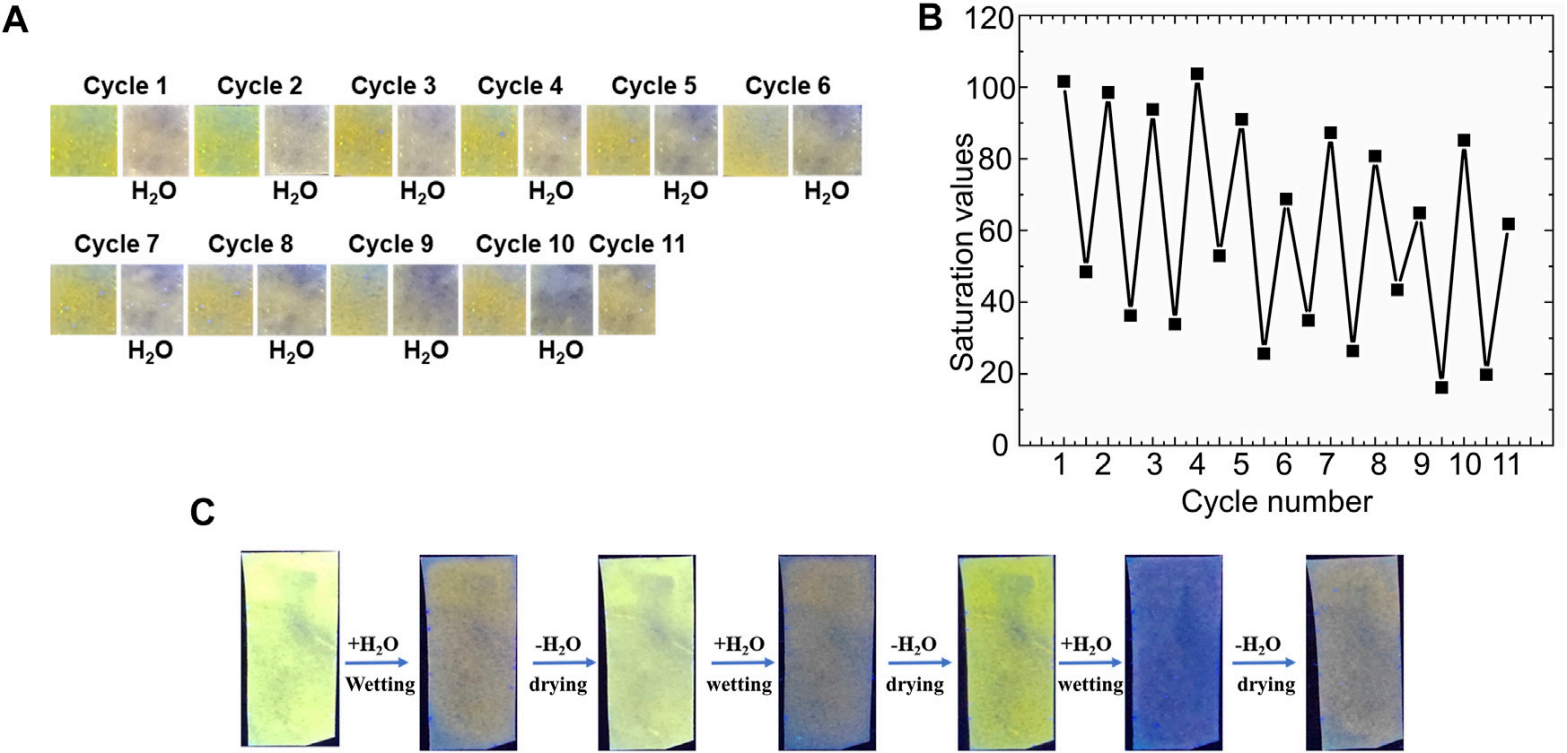
An illustration of the regeneration and reusability of bimane **1**-functionalized filter papers: **(A)** The detection of liquid water droplets over 11 cycles of exposure followed by drying; **(B)** Quantitative green values of the papers plotted as a function of cycle number, with each cycle representing an exposure to water followed by drying; and **(C)** Reusability of the paper sensors for the detection of humidity through exposing the papers to 99% relative humidity, followed by drying under ambient humidity conditions and re-exposure.

Finally, the bimane **1**-functionalized filter papers demonstrated strong reversibility in the sensing of water in both the liquid and vapor phase. Liquid water, which induced noticeable photophysical changes of the bimane **1**-functionalized paper (see Figure 6A, above), was effectively removed by drying the papers under ambient conditions followed by brief heating (80 °C for 10 min). This drying allowed for the introduction of water again to the paper, followed by re-drying of the paper under the aforementioned conditions. Overall, 11 cycles of sensing followed by drying were demonstrated, albeit with progressive decreases in the performance observed. This reusability was visible by naked eye detection (Figure 7A), and could also be quantified by measuring the saturation values for each of the papers (Figure 7B). Similarly, the reusability of bimane **1**-functionalized papers as humidity sensors was also demonstrated, with 7 cycles of exposure to 99% relative humidity, followed by drying and reuse of the same sensor with comparable performance results (Figure 7C). Of note, at this stage we cannot say with full certainty that in the dry state the original bimane **1** is regenerated. Reaction of bimane **5** with free OH groups in the surface of the paper would also lead to fluorescent bimane based boronate esters, which means that the observed partial reversibility may be a result of the generation of new fluorescent bimane-based boronate esters on the paper support.

## Conclusion

The development of methods for the rapid, sensitive, and generally applicable detection of water represents a high priority research area due to its significant applications in chemical safety and industry. The work reported herein represents an important step towards addressing this issue, by reporting that a boronate ester-functionalized bimane, compound **1**, undergoes rapid and sensitive fluorescence quenching in the presence of trace quantities of water, both in solution and in the vapor phase. The successful access to this structure due to recent advances in synthetic methodology for *syn*-bimanes represents an additional important advance, and the reusability of paper-based sensors with compound **1** highlights the strong practical applicability of this system. Future work will be directed towards demonstrating the broad applicability of this sensor in real-world samples, and results of these and other investigations will be reported in due course.

## Data Availability

The original contributions presented in the study are included in the article/Supplementary Material and further inquiries can be directed to the corresponding authors.
