# Supplementary material for: Highly Sensitive Water Detection Through Reversible Fluorescence Changes in a syn-Bimane Based Boronic Acid Derivative
Source: Front Chem. 2022 Jan 17;9:782481. doi: 10.3389/fchem.2021.782481 (PMC8802138; doi:10.3389/fchem.2021.782481)
Supplement: Supplementary file 1 [file DataSheet1.PDF]

Electronic Supporting Information for

Highly Sensitive Water Detection through Reversible Fluorescence Changes in a *syn*-Bimane Based Boronic Acid Derivative

Apurba Pramanik†, Joy Karmakar†, Flavio Grynszpan*, and Mindy Levine*

† These authors contributed equally to this work

* Authors to whom the correspondence should be addressed. flaviog@ariel.ac.il and mindyl@ariel.ac.il

TABLE OF CONTENTS

Materials and Methods……S3

Synthesis of Bimane **1**……S4

Experimental Procedures……S6

Experimental Procedures for Solution-State UV-Visible and Fluorescence Studies……S6

Experimental Procedures for Limit of Detection Studies……S7

Experimental Procedure for pH-Dependent Colorimetric Studies……S8

Experimental Procedures for Paper-Based Studies……S9

Experimental Procedures for ^{1}H NMR Titration Studies……S9

Experimental Procedures for High Resolution Mass Spectrometry Studies……S9

Experimental Procedures for Colorimetric Solution-State Studies……S9

Experimental Procedures for Colorimetric Solid-State Studies……S9

Experimental Procedures for Kinetics Investigations……S9

Experimental Procedures for Reversibility Studies……S10

Experimental Procedure for the Determination of Relative Quantum Yield……S10

Experimental Procedure for Calculation of the Molar Extinction Coefficient……S10

Summary Tables……S11

Summary Tables for UV-Visible and Fluorescence Studies……S11

Summary Tables for Limit of Detection Studies……S16

Summary Tables for Solution-State Colorimetric Studies……S17

Summary Tables for Solid-State Studies……S21

Summary Tables for ^{1}H NMR Titration Studies……S22

Summary Tables for Kinetic Studies……S24

Summary Figures……S25

Summary Figures from Bimane Synthesis……S25

Summary Figures for UV-Visible and Fluorescence Studies……S27

Summary Figures for Limit of Detection Studies……S35

Summary Figures for Solution State Studies……S43

Summary Figures for Solid-State Studies……S54

Summary Figures for ^{1}H NMR Titration Studies……S55

Summary Figures for High Resolution Mass Spectrometry Studies……S58

Summary Figures for Kinetic Studies……S59

Summary Figures for Solid-State Reversibility Studies……S60

Summary Figures for Quantum Yield Experiments……S63

Summary Figures for pH Dependent Experiments……S64

References……S65

MATERIALS AND METHODS

All chemicals, including spectroscopic grade solvents, were purchased from commercial suppliers and used without further purification. All UV/Visible absorption spectra were recorded on a Varian Cary 50 Bio UV-visible spectrophotometer. Fluorescence spectra were recorded on a Varian Cary Eclipse fluorescence spectrophotometer, with emission recorded at a 90° angle relative to the excitation. ^{1}H NMR spectra were obtained using a Bruker Avance III spectrophotometer operating at 400 MHz. The pH values of aqueous solutions were recorded on a Mettler Toledo Education Line pH meter. All fluorescence spectra were integrated vs. wavenumber on the X-axis using OriginPro 2020. All curve fitting was done using OriginPro curve fitting options (either linear or non-linear curve fitting, as applicable). All the HR-MS spectra were measured in positive ionization mode using a Waters Micromass Quattro Micro instrument, which was equipped with an electrospray ionization source with Waters 2795 and 996 PDA detectors.

SYNTHESIS OF BIMANE 1

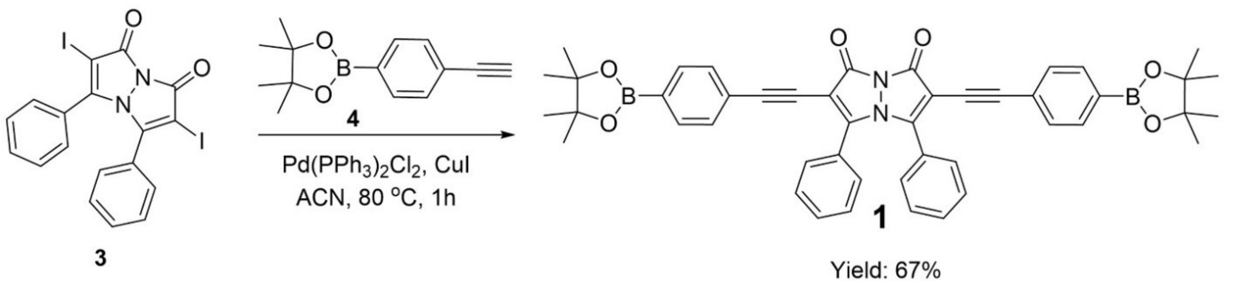

Figure S1. Synthesis of compound **1**

Compound **1** was synthesized from compounds **3** and **4**. Bis(triphenylphosphine)palladium (II) chloride (20 mg, 0.028 mmol, 0.10 equiv) and cuprous iodide (2.7 mg, 0.014 mmol, 0.05 equiv) were added to a solution of 4-ethynylphenylboronic acid pinacol ester **4** (140 mg, 0.61 mmol, 2.2 equiv), diisopropylethylamine (0.48 mL, 2.8 mmol, 10 equiv), and compound **3** (150 mg, 0.28 mmol, 1.0 equiv) in CH_3CN (200 mL). The mixture was stirred at 80 °C for one hour under a nitrogen atmosphere. After one hour, the solvent was evaporated under reduced pressure, and the resulting crude product was purified via flash chromatography over silica gel eluting with 5% ethyl acetate: 95% dichloromethane. The product was isolated as a reddish-yellow colored solid in 67 % yield (138 mg). ^{1}H NMR ($CDCl_3$): 7.72-7.70 (*d*, *J* = 8 Hz, 2 H, Ar-H), 7.36-7.34 (*d*, *J* = 8 Hz, 2 H, Ar-H), 7.32-7.28 (m, 2 H, Ar-H), 7.24-7.23 (*d*, *J* = 4 Hz, 1 H, Ar-H), 7.17-7.13 (*m*, 2 H, Ar-H), 1.33 (*s*, 12 H, 2(-Me)$_2$) ppm; ^{13}C NMR ($CDCl_3$): 134.64, 131.24, 130.89, 129.31, 128.29, 84.15, 25.01 ppm; DEPTQ: 134.96, 131.20, 129.63, 128.61, 126.12, 25.32 ppm; HRMS *m/z*: $[M+H]^+$ calculated: 741.3322, found: 741.3332.

A number of options, which are summarized in Figures S2-S4, were explored for the synthesis of compound **3**. In brief, β–ketoester **S1** was converted into compound **2** in high yield, following by dichlorination to access compound **S2**. This halogenation process occurred either using chlorine gas in dichloromethane (40% isolated yield),[1] or via an improved process using trichloroisocyanuric acid (TCCA), according to previous reports from our research group (54% isolated yield).[2] Treatment with potassium carbonate in dichloromethane led to a mixture of the desired bimane product **S4** (26% yield) and undesired *anti* isomer **S3** (5% yield). Compound **S4** was subsequently converted into compound **3** using a two-step procedure in high yield (Figure S3). Alternatively, compound **2** could be converted directly into compound **3** using an adaption of our recently reported one-pot procedure (Figure S4).[3] This option provided access to compound **3** in 68% overall yield.

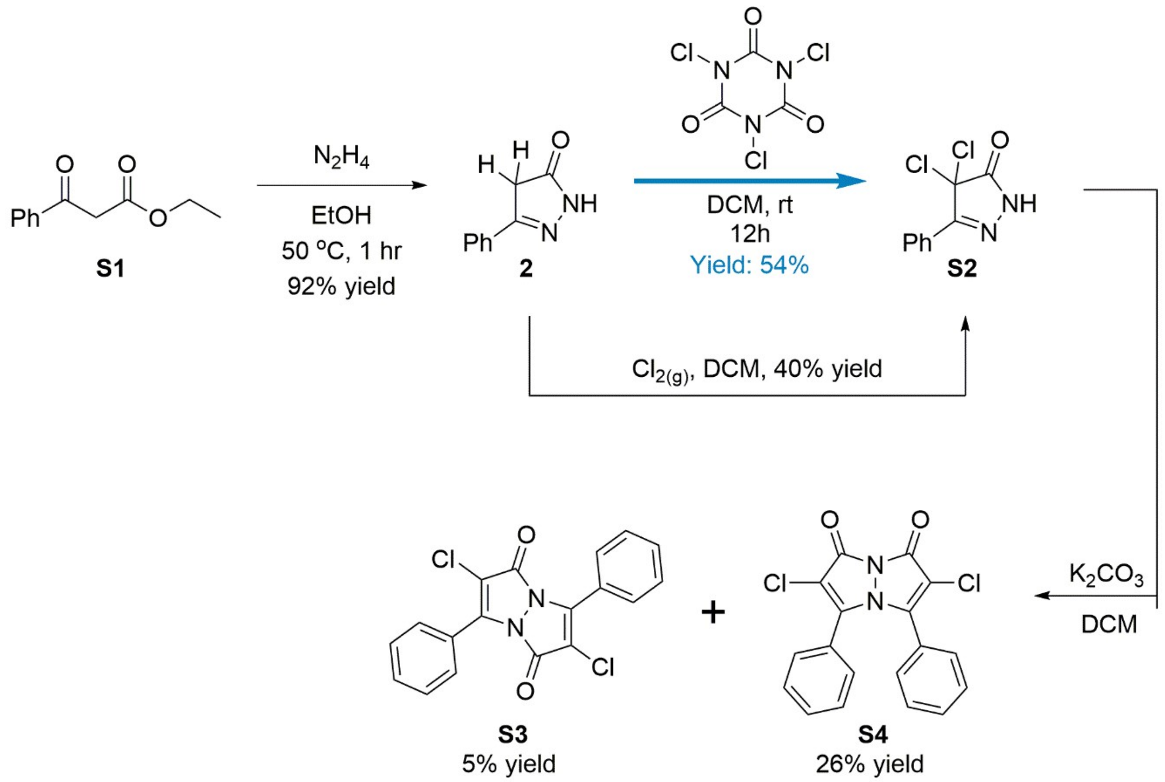

Figure S2. Options explored to access compound **S4**, a key intermediate in the synthesis of bimane **1**.

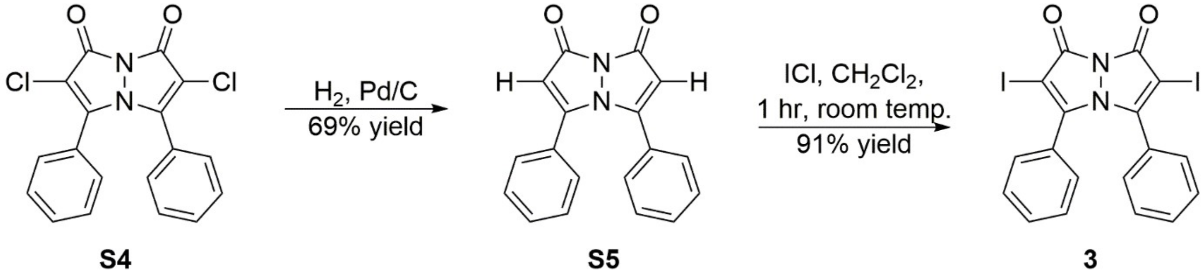

Figure S3. The conversion of compound **S4** into compound **3**.

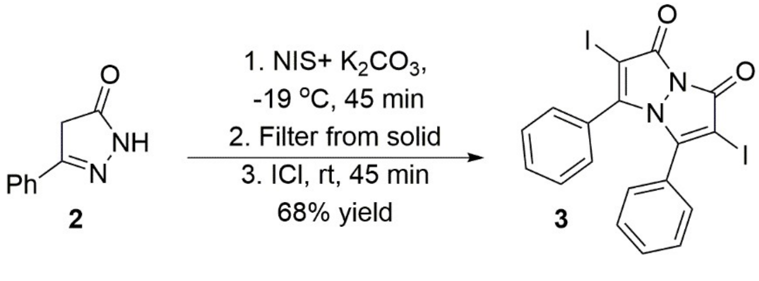

Figure S4. A streamlined method for the conversion of compound **2** into compound **3** in 68% overall yield.

EXPERIMENTAL PROCEDURES

Experimental Procedures for Solution-State UV-Visible and Fluorescence Spectroscopy

Experimental Procedures for UV-Vis Absorption Spectrometry

UV-Visible absorption spectroscopy was used in two different situations: (a) to study the solvent dependent properties of the bimane-based boronate compound **1**; and (b) to investigate the detection of water using bimane-based boronate compound **1**. Procedures for each of these situations are detailed below.

(a) To study the solvent dependent properties of compound **1**:

The effects of 13 different solvent systems were investigated by measuring the UV-visible absorption spectra of bimane **1** (from 200-700 nm) at a concentration of 10 µM in each of the solvent systems (see Table S1**)**.

(b) To investigate the detection of water using compound **1**:

The changes of the UV-visible absorbance spectrum of compound **1** upon the addition of varying concentrations of water was measured in a variety of water-miscible solvents. These experiments were conducted in HPLC grade solvents, with the concentration of compound **1** held constant at 10 µM, and with increasing concentrations of Milli-Q purified water added to the solution.

Experimental Procedure for Fluorescence Spectroscopy

Fluorescence spectroscopy was used to investigate two different situations: (a) to study the solvent dependent fluorescence properties of compound **1**; and (b) to investigate the detection of water using compound **1** in a variety of solvents. In all cases, the excitation of bimane **1** occurred at 450 nm, and the excitation and emission slit widths were both 5.0 nm. The procedures used in each of these situations are discussed in detail below:

(a) To study the solvent dependent fluorescence properties of compound **1**:

The concentration of compound **1** was held constant at 10 µM in acetonitrile, acetonitrile-water (1:1 vol: vol), methanol, ethanol, tetrahydrofuran, ethyl acetate, dichloromethane, chloroform, acetone, diethyl ether, *N,N*-dimethylformamide (DMF), dimethylsulfoxide (DMSO), and water. The fluorescence emission of compound **1** in each solvent was recorded via excitation at 450 nm. Changes in the fluorescence emission of bimane **1** were quantified by integrating the fluorescence emission vs. wavenumber on the X-axis (using OriginPro 2020).

(b) To investigate the detection of water using compound **1**:

Increasing concentrations of water were added to solutions of compound **1** in organic, water-miscible solvents, with the concentration of bimane **1** held constant at 10 µM. The concentration of water in these experiments ranged from 0 µM to 43.16 µM. In all cases, changes in the fluorescence emission of compound **1** under these conditions were quantified by integrating the fluorescence emission vs. wavenumber on the X-axis using OriginPro 2020.

Experimental Procedures for Limit of Detection Studies

Fluorescence-Based Limit of Detection

The experimental procedures used for limit of detection studies followed literature-reported procedures.[4] These procedures are also reviewed below:

1. 100 µL of a solution of compound **1** (0.1 mg/mL) in different solvents (*N,N*-dimethylformamide, dimethylsulfoxide, tetrahydrofuran, or acetonitrile) were measured into a 15 mL glass vial. 2.40 mL of the same solvents were added to dilute the stock solution.
2. The diluted solution was transferred to a quartz cuvette and then excited at 450 nm and the fluorescence emission spectra was recorded from 460 to 800 nm. Each fluorescence measurement was repeated six times.
3. 5.0 µL of Milli-Q water was added to the cuvette and stirred thoroughly to ensure homogeneity. The solution was excited at the same wavelength (450 nm) and the emission was measured between 460 nm and 800 nm. Six repeat measurements were taken.
4. Step 2 was repeated four times for total addition volumes of 10 µL, 15 µL, 20 µL, and 25 µL of Milli-Q water. In all cases, the solution was excited at 450 nm and the emission spectra from 460 to 800 nm was recorded six times.
5. For each solvent, a graph was plotted with integrated emission of compound **1** (vs. wavenumber) on the Y-axis and concentration of water (in µM) on the X-axis, and an equation for the straight line was determined. The limit of detection of the blank sample (LOD_{blank}) was defined according to the following equation:

   $$LOD_{blank} = m_{blank} + 3(SD_{blank}) \qquad \text{(Eq. S1)}$$

   where m_{blank} is the mean of the blank integrations and SD_{blank} is the standard deviation of those measurements.
6. The LOD_{blank} was then entered into the equation determined from the plot of integrated emission vs concentration of water, and the corresponding X-value was calculated. This value is the limit of detection of the analyte in µM.
7. The limit of quantification of the blank sample (LOQ_{blank}) was calculated in a similar manner to the LOD. The limit of quantification of the blank is defined according to the following equation:

   $$LOQ_{blank} = m_{blank} + 10(SD_{blank}). \qquad \text{(Eq. S2)}$$
8. This value is then entered as the y-value from the derived equation, and the corresponding X-value was calculated. This is the value of the limit of quantification (LOQ) of the analyte for the system in µM.

Colorimetric-Based Limit of Detection

The experimental procedures used for limit of detection studies based on colorimetric data were similar to those reported above for fluorescence-based data, with the following key modifications:

1. The amounts of water added to the solutions of bimane **1** in water-miscible solvents were: 0 μM, 74 μM, 148 μM, 222 μM, 296 μM, and 370 μM.
2. Colorimetric values were determined by using the photograph of the solutions under excitation by long-wave TLC lamp irradiation (at 365 nm), and quantifying the red, green, and blue values of each solution using ImageJ software.
3. Six values for each red, green, and blue value were obtained by cropping different segments of the solutions' photographs and measuring the RGB values of each cropped segment.
4. The limits of detection of the blank sample and limit of quantification of the blank sample were calculated in the same manner as discussed above.
5. The calibration graphs were constructed with the concentration of water (in μM) on the X-axis, and the red, green, or blue value of the solution on the Y-axis. The best linear fit for each graph was determined, and the line was determined to be a valid representation of the data in cases where the R^2 value was above 0.90.
6. The limits of detection of the blank and limits of quantification of the blank were used as the Y-value in the best-fit linear equation, and the X value corresponding to those Y values represented the limit of detection and limit of quantification, respectively.

Experimental Procedure for pH-Dependent Colorimetric Studies

Colorimetric solution-state studies were done under different pH condition. 10% (v/v) Phosphate buffer solutions in acetonitrile were added ([**1**] = 10 μM). A photograph of each solution was taken both in ambient light and under excitation by a hand-held, long-wave TLC lamp at 365 nm.

Experimental Procedures for Paper-Based Studies

Whatman #1 filter papers with dimensions of 3.5 cm x 0.9 cm were coated with a solution of compound **1** by submerging the filter papers in an acetonitrile solution of compound **1** ([**1**] = 10 µM) for sixty minutes at room temperature. After 60 minutes, the papers were carefully removed from the solution using tweezers and were then placed on a Petri dish and allowed to dry for three hours in an open-air environment. After that, varying amounts of water (100, 150, 200, 250, 300, or 350 µL) were added via pipette to the top of the paper, and the paper was allowed to dry for four hours at room temperature on a benchtop. The dried papers were then visualized under a long-wave, hand-held TLC lamp (365 nm excitation) and the results of these studies are reported herein.

Experimental Procedures for ^{1}H NMR Titration Studies

Approximately 2.0 mg of bimane **1** were dissolved in 450 µL of DMSO-d_6, and the ^{1}H NMR spectrum was recorded. Then we added 30 µL of D_2O in the NMR tube and vortexed, the ^{1}H NMR spectrum was recorded a second time. We continued to increase the amount of water in increments of 30 µL, recording the ^{1}H NMR spectrum after each addition, up to a total addition of 210 µL of D_2O.

Experimental Procedures for High Resolution Mass Spectrometry Studies

We prepared 10 µM bimane **1** solution in an acetonitrile-water (1:1) mixture and then collected the HRMS data using a Waters Micromass Quattro micro instrument, which was equipped with an electrospray ionization source with Waters 2795 and 996 PDA detectors. We then did a second experiment, but replaced the acetonitrile-water solvent mixture with a 100% methanol solvent. The HRMS spectrum was recorded again.

Experimental Procedures for Colorimetric Solution-State Studies

Colorimetric solution-state studies were done in two different situations: (a) to measure the differences in the color of the solution of bimane **1** in various solvents; and (b) to measure changes in the color of the solutions of bimane **1** in various water-miscible solvents that occurred as a result of the addition of water. These colorimetric changes were quantified using the following procedure:

1. A photograph of each one of the solutions was taken both in ambient lighting and under excitation by a hand-held, long-wave TLC lamp;
2. The resulting images were cropped using Microsoft Paint so that only the solution was visible; and
3. The RGB values were quantified using ImageJ software (https://imagej.nih.gov/ij/) and/or with Microsoft Paint using a random sampling of at least 10 data points.

Experimental Procedures for Colorimetric Solid-State Studies

Colorimetric solid-state studies were done to measure the differences in the color of the bimane-functionalized filter paper upon exposure to different amounts of water. The resulting colorimetric changes that occurred from such exposure were quantified using the following procedure:

1. A photograph of the filter paper was taken both in ambient lighting and under excitation by a hand-held TLC lamp (excitation at 365 nm???);
2. The resulting images were cropped using Microsoft Paint so that only the functionalized paper was visible; and
3. The RGB values were quantified using ImageJ software (https://imagej.nih.gov/ij/).

Experimental Procedures for Kinetics Investigations

We investigated the time-dependent decrease in the fluorescence signal of bimane **1** in an acetonitrile-water (1:1 vol: vol) solution; ([**1**] = 10 µM), via excitation of the solution at 450 nm and excitation and emission slit widths of 5.0 nm. We recorded the fluorescence emission spectrum at two-minute time intervals between 0 and 50 minutes. Changes in the fluorescence intensity were quantified by integrating the emission spectra vs. wavenumber on the X-axis using OriginLab 2021, and the best linear fit between the integrated fluorescence emission (on the Y-axis) and time of the experiment (on the X-axis) provided information about the kinetics of the water detection process.

Experimental Procedures for Reversibility Studies

Water Reversibility Studies: Whatman #1 filter papers with dimensions of 1.0 cm x 0.8 cm were coated with a solution of bimane **1** ([**1**] = 10 μM) in acetonitrile by submerging the filter papers in the solution for sixty minutes at room temperature. After sixty minutes, the papers were carefully removed from the solution using tweezers and were then placed on a Petri dish and allowed to dry overnight in an open-air environment, and then dried at 80 ^{O}C for ten minutes. These papers were imaged using a TLC lamp with 365 nm excitation. After that, we added 1 drop of milli-Q water by using a 20-200 μL pipette. An image of the paper under the same excitation wavelength (365 nm) was then acquired. Then the paper was allowed to dry on the benchtop for 45 min and at 80 ^{O}C for 10 min, followed by re-imaging. The same cycle of adding one drop of water, followed by drying at ambient atmosphere for 45 minutes and then at 80 ^{O}C for ten minutes were repeated for several cycles, and the results of these investigations are reported herein.

Humidity Reversibility Studies: Whatman #1 filter papers with dimensions of 3.0 cm x 0.95 cm were coated with a solution of bimane **1** ([**1**] = 200 μM) in acetonitrile by submerging the filter papers in the solution for sixty minutes at room temperature. After sixty minutes, the papers were carefully removed from the solution using tweezers and were then placed on a Petri dish and allowed to dry overnight in an open-air environment. These papers were imaged using a TLC lamp with 365 nm excitation. After that, the paper was kept in a humid chamber (99.9% relative humidity) for 4 hours to allow for the absorption of water. An image of the paper under the same excitation wavelength (365 nm) was then acquired. The paper was allowed to dry on the benchtop for 4-5 hours, followed by re-imaging. The same cycle of exposure to a humid chamber for 4 hours followed by exposure to ambient atmosphere for 4-5 hours was repeated for several cycles, and the results of these investigations are reported herein.

Humidity Chamber Construction: A high humidity chamber was constructed from a glass beaker filled with tissue paper that had been soaked in Milli-Q water and covered with parafilm. The relative humidity in the chamber was measured at 99.9% using an eSynic Thermometer and Hygrometer digital LCD display instrument. These experiments were conducted at an ambient air temperature of 27 °C and an ambient humidity of 54%.

Experimental Procedure for the Determination of Relative Quantum Yield:

The fluorescence spectra for calculating the quantum yield of bimane **1** in acetonitrile were recorded using a Varian Cary Eclipse fluorescence spectrophotometer equipped with a 10 mm pathlength quartz cuvette. The excitation slit width was 5.0 nm, the emission slit width was 5.0 nm, and the scan rate was 120 nm/min. Quantum yields were calculated using the single point relative quantum yield method, according to Equation S3, below:[5]

$$\phi_{F(x)} = (n_x/n_s)^2 (A_s/A_x)(I_{f(x)}/I_{f(s)})\phi_{F(s)} \quad \text{(Eq. S3)}$$

where ϕ_F represents the fluorescence quantum yield, A represents the absorbance value, I_f represents the integration of the fluorescence band, and n represents the solvent refractive index. The subscripts s and x refer to the standard and unknown sample, respectively. In this work, the fluorescence quantum yield standard was a solution of 1,4-bis(5-phenyloxazol-2-yl)benzene (POPOP) in cyclohexane, with a reported quantum yield of 0.97.[6]

Experimental Procedure for Calculation of the Molar Extinction Coefficient

For the calculation of the molar extinction coefficient of bimane **1** we used the Beer-Lambert equation S4:

$$A = \varepsilon * c * l \quad \text{(Eq. S4)}$$

where A represents the absorbance of the solution at a particular wavelength, ε represents the molar extinction coefficient, c represents the concentration (mol/L) and l represents the path length (cm). In this calculation, we have measured the absorbance value of 0.1227 at 450 nm wavelength. Based on this data, the calculated molar extinction coefficient is 1.23 x 10^4 L M^{-1} cm^{-1}.

SUMMARY TABLES

Summary Tables for UV-Visible and Fluorescence Studies

Table S1. Results of the UV-Visible Absorption and Fluorescence Emission Spectra of Bimane **1** in Various Solvents[a]

Solvent	Maximum absorbance value above 350 nm	Wavelength at which maximum absorbance occurs	Normalized integrated fluorescence emission
Acetonitrile	0.12 ± 0.000	449.0 ± 1.4	0.75 ± 0.001
Acetonitrile-water (1:1)	0.15 ± 0.000	455.0 ± 0.0	0.33 ± 0.001
Methanol	0.12 ± 0.001	450.0 ± 0.0	0.33 ± 0.000
Ethanol	0.12 ± 0.000	449.5 ± 0.7	0.41 ± 0.000
Tetrahydrofuran	0.14 ± 0.000	448.5 ± 0.7	0.93 ± 0.000
Ethyl acetate	0.14 ± 0.002	446.0 ± 0.0	0.98 ± 0.006
Dichloromethane	0.11 ± 0.000	452.5 ± 2.1	0.62 ± 0.001
Chloroform	0.13± 0.000	453.0 ± 0.0	0.66 ± 0.001
Acetone	0.15 ± 0.000	448.0 ± 0.0	0.99 ± 0.000
Diethyl ether	0.13 ± 0.000	442.5 ± 0.7	1.00 ± 0.001
N,N-Dimethyl formamide (DMF)	0.11 ± 0.002	450.5 ± 2.1	0.58 ± 0.002
Dimethyl sulfoxide (DMSO)	0.12 ± 0.000	454.0 ± 0.0	0.59 ± 0.002
Water	0.07 ± 0.000	451.0 ± 0.0	0.03 ± 0.000

[a] Full spectrum UV-visible absorption spectra were measured on a Varian Cary 50 Bio UV-visible spectrophotometer, and fluorescence spectra were measured via excitation at 450 nm, followed by integration vs. wavenumber on the X-axis from 460 nm to 700 nm (21739 cm^{-1} to 14286 cm^{-1}). All results represent an average of at least 2 trials.

Table S2. Normalized Integration Values of the UV-Visible Absorption and Fluorescence Emission Spectra of Bimane **1** in Acetonitrile with the Addition of Water[a]

$[H_2O]$ (μM)	UV-vis absorption spectra	Fluorescence emission spectra
0	1.00 ± 0.000	1.00 ± 0.001
1.66	0.99 ± 0.000	0.94 ± 0.001
3.32	0.98 ± 0.001	0.89 ± 0.000
4.98	0.97 ± 0.000	0.84 ± 0.000
6.64	0.96 ± 0.001	0.80 ± 0.000
8.30	0.95 ± 0.000	0.76 ± 0.000
9.96	0.94 ± 0.000	0.72 ± 0.000

11.62	0.93 ± 0.002	0.70 ± 0.000
13.28	0.92 ± 0.001	0.67 ± 0.000
14.94	0.91 ± 0.000	0.64 ± 0.000
16.60	0.91 ± 0.001	0.62 ± 0.000
18.26	0.90 ± 0.002	0.60 ± 0.000
19.92	0.89 ± 0.000	0.58 ± 0.000
21.58	0.88 ± 0.001	0.56 ± 0.000
23.24	0.87 ± 0.001	0.55 ± 0.000
24.90	0.87 ± 0.001	0.53 ± 0.000
26.56	0.86 ± 0.003	0.52 ± 0.000
28.22	0.85 ± 0.002	0.51 ± 0.000
29.88	0.84 ± 0.001	0.50 ± 0.000
31.54	0.84 ± 0.001	0.48 ± 0.000
33.20	0.84 ± 0.001	0.48 ± 0.000
34.86	0.82 ± 0.002	0.47 ± 0.000

[a] Absorption spectra were integrated between 365 and 545 nm. Fluorescence spectra were integrated over the full spectrum (460-700 nm). All spectra were integrated vs. wavenumber on the X-axis, and the results reported herein represent the average of at least two trials.

Table S3. Normalized Integration Values of the UV-Vis Absorption and Fluorescence Emission Spectra of Bimane **1** in Tetrahydrofuran with the Addition of Water[a]

$[H_2O]$ (μM)	UV-vis absorption spectra	Fluorescence emission spectra
0	1.00 ± 0.001	1.00 ± 0.001
1.66	0.99 ± 0.000	0.95 ± 0.001
3.32	0.98 ± 0.002	0.90 ± 0.001
4.98	0.97 ± 0.001	0.86 ± 0.001
6.64	0.96 ± 0.001	0.83 ± 0.001
8.30	0.96 ± 0.001	0.81 ± 0.001
9.96	0.95 ± 0.002	0.79 ± 0.001
11.62	0.95 ± 0.003	0.76 ± 0.000
13.28	0.94 ± 0.001	0.75 ± 0.001
14.94	0.94 ± 0.003	0.72 ± 0.000
16.60	0.94 ± 0.001	0.71 ± 0.000
18.26	0.94 ± 0.003	0.69 ± 0.000
19.92	0.94 ± 0.002	0.67 ± 0.000

21.58	0.93 ± 0.000	0.66 ± 0.000
23.24	0.93 ± 0.004	0.64 ± 0.000
24.90	0.93 ± 0.002	0.63 ± 0.001
26.56	0.93 ± 0.007	0.62 ± 0.000
28.22	0.93 ± 0.001	0.60 ± 0.000
29.88	0.93 ± 0.000	0.60 ± 0.000
31.54	0.93 ± 0.007	0.59 ± 0.000

[a] Absorption spectra were integrated between 365 and 545 nm. Fluorescence spectra were integrated over the full spectrum (460-700 nm). All spectra were integrated vs. wavenumber on the X-axis, and the results reported herein represent the average of at least two trials.

Table S4. Normalized Integration Values of the UV-Vis Absorption and Fluorescence Emission Spectra of Bimane **1** in *N,N*-Dimethylformamide with the Addition of Water[a]

$[H_2O]$ (μM)	UV-vis absorption spectra	Fluorescence Emission Spectra
0	0.99 ± 0.014	1.00 ± 0.004
1.66	0.93 ± 0.001	0.97 ± 0.001
3.32	0.93 ± 0.000	0.95 ± 0.000
4.98	0.92 ± 0.001	0.92 ± 0.000
6.64	0.91 ± 0.003	0.90 ± 0.000
8.30	0.90 ± 0.005	0.87 ± 0.000
9.96	0.89 ± 0.000	0.85 ± 0.000
11.62	0.88 ± 0.008	0.83 ± 0.000
13.28	0.87 ± 0.002	0.81 ± 0.000
14.94	0.86 ± 0.000	0.79 ± 0.001
16.60	0.85 ± 0.000	0.77 ± 0.000
18.26	0.85 ± 0.001	0.75 ± 0.000
19.92	0.84 ± 0.001	0.72 ± 0.000
21.58	0.84 ± 0.009	0.70 ± 0.001
23.24	0.82 ± 0.000	0.68 ± 0.000
24.90	0.82 ± 0.000	0.67 ± 0.001
26.86	0.81 ± 0.001	0.64 ± 0.000
28.22	0.80 ± 0.002	0.63 ± 0.001
29.88	0.80 ± 0.002	0.60 ± 0.001
31.54	0.78 ± 0.001	0.59 ± 0.000
33.20	0.78 ± 0.003	0.57 ± 0.001

34.86	0.78 ± 0.008	0.55 ± 0.000
36.52	0.77 ± 0.007	0.54 ± 0.001
38.18	0.76 ± 0.003	0.52 ± 0.000
39.84	0.75 ± 0.001	0.51 ± 0.001
41.50	0.75 ± 0.005	0.51 ± 0.001
43.16	0.74 ± 0.001	0.50 ± 0.001

[a] Absorption spectra were integrated between 365 and 545 nm. Fluorescence spectra were integrated over the full spectrum (460-700 nm). All spectra were integrated vs. wavenumber on the X-axis, and the results reported herein represent the average of at least two trials.

Table S5. Normalized Integration Values of the UV-Vis Absorption and Fluorescence Emission Spectra of Bimane **1** in Acetone with the Addition of Water[a]

[H_2O] (μM)	UV-vis absorption spectra	Fluorescence Emission Spectra
0	1.00 ± 0.000	1.00 ± 0.002
1.66	0.99 ± 0.000	0.95 ± 0.000
3.32	0.98 ± 0.000	0.90 ± 0.00
4.98	0.97 ± 0.000	0.87 ± 0.000
6.64	0.97 ± 0.000	0.83 ± 0.000
8.30	0.96 ± 0.000	0.80 ± 0.000
9.96	0.95 ± 0.001	0.77 ± 0.000
11.62	0.94 ± 0.000	0.74 ± 0.000
13.28	0.94 ± 0.000	0.72 ± 0.000
14.94	0.93 ± 0.001	0.70 ± 0.000
16.60	0.92 ± 0.000	0.68 ± 0.000
18.26	0.92 ± 0.001	0.66 ± 0.000
19.92	0.91 ± 0.001	0.64 ± 0.000
21.58	0.90 ± 0.000	0.63 ± 0.000
23.24	0.90 ± 0.000	0.61 ± 0.000
24.90	0.89 ± 0.001	0.60 ± 0.000
26.86	0.89 ± 0.000	0.59 ± 0.000
28.22	0.88 ± 0.001	0.57 ± 0.000
29.88	0.87 ± 0.001	0.56 ± 0.000
31.54	0.87 ± 0.002	0.55 ± 0.000
33.20	0.86 ± 0.000	0.54 ± 0.000

[a] Absorption spectra were integrated between 365 and 545 nm. Fluorescence spectra were integrated over the full spectrum (460-700 nm). All spectra were integrated vs. wavenumber on the X-axis, and the results reported herein represent the average of at least two trials.

Table S6. Normalized Integration Values of the UV-Vis Absorption and Fluorescence Emission Spectra of Bimane **1** in Dimethylsulfoxide (DMSO) with the Addition of Water[a]

[H_2O] (μM)	UV-vis absorption spectra	Fluorescence Emission Spectra
0	0.98 ± 0.000	1.00 ± 0.003
1.66	1.00 ± 0.000	0.99 ± 0.000
3.32	0.99 ± 0.000	0.97 ± 0.000
4.98	0.99 ± 0.003	0.96 ± 0.001
6.64	0.97 ± 0.001	0.95 ± 0.000
8.30	0.97 ± 0.002	0.93 ± 0.000
9.96	0.96 ± 0.000	0.92 ± 0.000
11.62	0.95 ± 0.002	0.90 ± 0.000
13.28	0.94 ± 0.000	0.89 ± 0.000
14.94	0.93 ± 0.004	0.87 ± 0.001
16.60	0.93 ± 0.009	0.86 ± 0.000
18.26	0.92 ± 0.000	0.85 ± 0.000
19.92	0.91 ± 0.000	0.83 ± 0.000
21.58	0.90 ± 0.002	0.81 ± 0.000
23.24	0.89 ± 0.001	0.80 ± 0.000
24.90	0.89 ± 0.014	0.79 ± 0.000
26.56	0.88 ± 0.010	0.77 ± 0.000
28.22	0.88 ± 0.007	0.76 ± 0.000
29.88	0.86 ± 0.001	0.74 ± 0.000
31.54	0.86 ± 0.003	0.73 ± 0.000
33.20	0.84 ± 0.002	0.72 ± 0.000
34.86	0.84 ± 0.002	0.71 ± 0.000
36.52	0.83 ± 0.001	0.71 ± 0.000

[a] Absorption spectra were integrated between 365 and 545 nm. Fluorescence spectra were integrated over the full spectrum (460-700 nm). All spectra were integrated vs. wavenumber on the X-axis, and the results reported herein represent the average of at least two trials.

Summary Tables for Limit of Detection Studies

Fluorescence-Based Limit of Detection

Table S7. Summary of Limits of Detection and Quantification of Water in Various Water-Miscible Organic Solvents using Integrated Fluorescence Emission[a]

Solvent	Equation	R^2	Limit of Detection (v/v)	Limit of Quantification (v/v)
DMF	y = -20279x + 910013	0.9987	0.17 ± 0.007%	0.60 ± 0.014%
DMSO	y = -8197.5x + 820434	0.9804	0.018 ± 0.00%	0.19 ± 0.007%
Acetonitrile	y = -16944x + (1E6)	0.9808	0.57 ± 0.003%	0.96 ± 0.007%
THF	y = -17448x + 1E+06	0.9856	5.74 ± 0.014%	6.05 ± 0.014%
Acetone	y = -139585x + 1E+07	0.9979	0.76 ± 0.001%	1.08 ± 0.002%

[a] Limits of detection and quantification were calculated using the procedures detailed above, and all results represent the average of at least three trials.

Colorimetric-Based Limit of Detection

Table S8. Summary of Limits of Detection and Quantification of Water in Various Water-Miscible Organic Solvents using Quantitative Colorimetric Data[a]

Solvent	RGB	Equation	R^2	Limit of Detection (v/v)	Limit of Quantification (v/v)
Acetonitrile	Red	y = 0.0934x + 118.61	0.9008	6.82%	28.10%
	Green	y = -0.0991x + 162.13	0.9781	9.66%	33.71%
	Blue	y = 0.1622x + 82.113	0.956	33.78%	114.12%
Acetone	Red	y = 0.1369x + 113.97	0.8922	[b]	[b]
	Green	y = -0.0833x + 176.57	0.8244	[b]	[b]
	Blue	y = 0.1191x + 82.933	0.8233	[b]	[b]
DMF	Red	y = 0.0811x + 137.42	0.9869	10.00%	35.87%
	Green	y = -0.049x + 183.38	0.9287	1.62%	41.68%
	Blue	y = 0.1387x + 79.139	0.8798	[b]	[b]
THF	Red	y = 0.1387x + 126.5	0.8878	[b]	[b]
	Green	y = -0.0223 + 192.64	0.664	[b]	[b]
	Blue	y = 0.1346x + 64.176	0.9884	34.18%	83.04%
DMSO	Red	y = 0.0684x + 140.13	0.9445	16.89%	58.98%
	Green	y = -0.0005 + 164.6	0.0005	[b]	[b]
	Blue	y = 0.1254x + 66.836	0.9798	31.08%	103.10%

[a] Limits of detection and quantification were calculated using the procedures detailed above.

[b] Limits of detection and limits of quantification could not be accurately calculated due to the lack of a linear relationship in this plot.

Summary Tables for Solution-State Colorimetric Studies

*Solution-State Colorimetric Changes of Bimane **1** in Different Solvent Systems*

Table S9. Quantitative red, green, and blue values of solutions of bimane **1** under ambient light and long-wave TLC lamp irradiation with a variety of solvent systems[a]

	UV			ambient light		
Solvent	R	G	B	R	G	B
acetonitrile	119	191	127	199	190	149
acetonitrile-water (1:1)	147	135	137	184	169	122
methanol	143	145	160	200	188	146
ethanol	165	189	176	202	191	152
THF	152	222	174	197	188	140
ethyl acetate	193	199	168	204	192	150
dichloromethane	179	225	163	204	195	153
chloroform	187	218	134	204	194	151
acetone	122	220	139	208	201	158
diethyl ether	57	218	139	193	186	140

[a] Data were obtained from photographs of the bimane **1** solution under ambient light and under long-wave TLC light irradiation (365 nm), with processing using ImageJ software and linear curve fitting using Microsoft Excel; [**1**] = 10 µM.

*Colorimetric Detection of Water with Bimane **1** in Different Solvent Systems*

Table S10. Quantitative red, green, and blue values of acetonitrile solutions of bimane **1** under ambient light and long-wave TLC lamp irradiation (365 nm) with increasing percentages of water[a]

	Ambient light			UV light (365 nm)		
water percent	R	G	B	R	G	B
0%	187	178	131	123	176	90
10%	189	178	130	157	169	80
20%	190	178	133	160	162	113
30%	195	184	141	153	152	128
40%	196	186	145	159	156	152
50%	198	189	150	155	152	175
60%	195	187	159	103	108	181
70%	193	184	160	55	70	178
80%	198	190	169	43	49	157
90%	197	188	165	75	55	172
100%	194	185	166	61	40	145

[a] Data were obtained from photographs of the bimane **1** solution under ambient light and under long-wave TLC light irradiation (365 nm), with processing using ImageJ software and linear curve fitting using Microsoft Excel; [**1**] = 10 µM.

Table S11. Quantitative red, green, and blue values of tetrahydrofuran solutions of bimane **1** under ambient light and long-wave TLC lamp irradiation with increasing percentages of water[a]

	Ambient light			UV light (365 nm)		
water percent	R	G	B	R	G	B
0%	191	185	140	116	192	80
10%	194	186	136	143	186	66
20%	186	178	129	159	194	100
30%	187	178	125	167	194	106
40%	184	176	131	163	188	140
50%	190	181	136	170	183	132
60%	194	184	142	170	172	139
70%	195	185	142	140	140	141
80%	198	188	145	157	163	147
90%	196	184	147	139	111	157
100%	198	187	157	74	44	117

[a] Data were obtained from photographs of the bimane **1** solution under ambient light and under long-wave TLC light irradiation (365 nm), with processing using ImageJ software and linear curve fitting using Microsoft Excel; [**1**] = 10 μM.

Table S12. Quantitative red, green, and blue values of acetone solutions of bimane **1** under ambient light and long-wave TLC lamp irradiation (365 nm) with increasing percentages of water

	Ambient light			UV light (365 nm)		
water percent	R	G	B	R	G	B
0%	184	176	85	143	212	23
10%	182	172	104	173	200	21
20%	174	163	120	148	167	100
30%	172	161	116	170	178	127
40%	178	167	124	170	166	128
50%	182	173	133	171	160	144
60%	182	175	149	109	110	169
70%	188	181	159	68	70	165
80%	187	179	158	63	52	137
90%	193	183	158	83	52	134
100%	200	191	167	71	43	125

[a] Data were obtained from photographs of the bimane **1** solution under ambient light and under long-wave TLC light irradiation (365 nm), with processing using ImageJ software and linear curve fitting using Microsoft Excel; [**1**] = 10 μM.

Table S13. Quantitative red, green, and blue values of DMF solutions of bimane **1** under ambient light and long-wave TLC lamp irradiation with increasing percentages of water

	Ambient light			UV light (365 nm)		
water percent	R	G	B	R	G	B
0%	174	163	119	141	173	95
10%	180	171	129	166	199	100
20%	174	164	123	173	188	96
30%	171	160	119	189	186	126
40%	179	166	125	191	178	147
50%	183	174	146	127	119	173
60%	187	179	154	104	107	204
70%	187	178	156	94	86	183
80%	189	180	158	85	67	157
90%	195	184	158	92	61	145
100%	200	188	159	71	46	131

[a] Data were obtained from photographs of the bimane **1** solution under ambient light and under long-wave TLC light irradiation (365 nm), with processing using ImageJ software and linear curve fitting using Microsoft Excel; [**1**] = 10 μM.

Table S14. Quantitative red, green, and blue values of DMSO solutions of bimane **1** under ambient light and long-wave TLC lamp irradiation (365 nm) with increasing percentages of water.

	Ambient light			UV light (365 nm)		
water percent	R	G	B	R	G	B
0%	180	169	110	167	185	27
10%	188	177	130	159	172	64
20%	180	168	127	177	180	95
30%	180	169	134	164	159	130
40%	197	188	162	99	104	177
50%	197	190	165	89	79	170
60%	193	185	161	95	81	185
70%	196	187	163	95	67	171
80%	203	193	167	90	54	147
90%	188	177	150	83	45	126
100%	194	183	158	76	44	133

[a] Data were obtained from photographs of the bimane **1** solution under ambient light and under long-wave TLC light irradiation (365 nm), with processing using ImageJ software and linear curve fitting using Microsoft Excel; [**1**] = 10 μM.

Table S15. Linear fitting of the changes in the blue value of bimane **1** solutions under long-wave TLC light with increasing percentages of water[a]

Solvent	Linear Range	Equation	R^2 value
Acetonitrile	0-60% water	y = 15.864x + 64.5	0.9509
THF	0-60% water	y = 115.75x + 71.518	0.9207
DMF	10-60% water	y = 198.15x + 73.953	0.9841
DMSO	0-40% water	y = 313.7x + 30.98	0.9897
Acetone	0-100% water[b]	y = -190.15x + 221.16	0.9249

[a] Data were obtained from photographs of the bimane **1** solution under long-wave TLC light irradiation (365 nm), with processing using random sampling of data points with Microsoft Paint (>10 points/sample) and linear curve fitting using Microsoft Excel; [**1**] = 10 μM

[b] Green values were used as the blue values gave a poor linear fit

Summary Tables for Solid-State Studies

Table S16. Quantitative colorimetric analysis of bimane **1** functionalized filter papers which were exposed to different volumes of water, measured before and after the addition of water[a]

	before H_2O addition			after H_2O addition		
	R	G	B	R	G	B
100 µL	198	180	172	198	192	191
150 µL	197	179	168	181	179	178
200 µL	208	188	175	190	186	184
250 µL	215	195	177	204	200	196
300 µL	193	182	169	128	148	162
350 µL	172	171	172	108	133	167

[a] Quantitative colorimetric analysis was done by cropping a photo of the image under long-wave UV light irradiation, followed by subjecting the cropped images to RGB analysis via ImageJ software.

Summary Tables for ^{1}H NMR Titration Studies

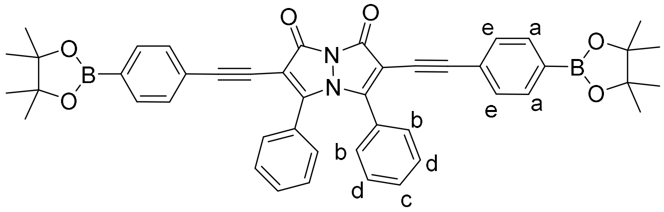

Table S17. Changes in the ppm values of aromatic proton peaks in the ^{1}H NMR spectrum of compound **1** in DMSO-d_6 upon the addition of varying amounts of D_2O[a]

Added D_2O (µL)	Ar-H_a	Ar-H_b	Ar-H_c	Ar-H_d	Ar-H_e
0 µL	7.720-7.700	7.657-7.637	7.289-7.255	7.208-7.159	7.359-7.338
30 µL	7.710-7.688	7.647-7.627	7.277-7.242	7.192-7.142	7.350-7.327
60 µL	7.700-7.683	7.636-7.616	7.267-7.237	7.180-7.130	7.344-7.317
90 µL	7.689-7.674	7.629-7.611	7.247-7.219	7.172-7.122	7.334-7.314
120 µL	7.688-7.669	7.624-7.604	7.242-7.211	7.159-7.112	7.331-7.311
150 µL	7.683-7.667	7.620-7.599	7.232-7.197	7.154-7.111	7.330-7.309
180 µL	7.682-7.651	7.617-7.597	7.229-7.186	7.150-7.098	7.291-7.270
210 µL	7.679-7.637	7.616-7.600	7.222-7.179	7.138-7.093	7.287-7.269

[a] ^{1}H NMR titrations were carried out following the procedures detailed above.

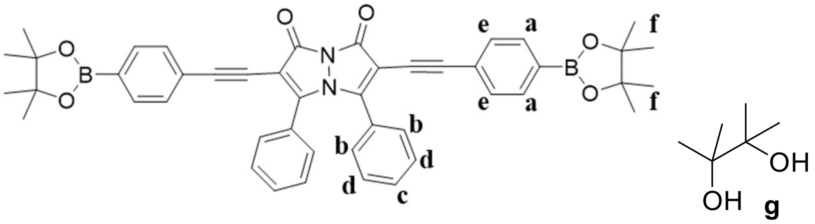

Table S18. Changes in the ppm values of aliphatic proton peaks in the ^{1}H NMR spectrum of compound **1** in DMSO-d_6 upon the addition of varying amounts of D_2O[a]

Added D_2O (µL)	H_f	H_g
0 µL	1.283	1.069
30 µL	1.265	1.056
60 µL	1.250	1.049
90 µL	1.240	1.046
120 µL	1.233	1.044
150 µL	1.227	1.042
180 µL	1.224	1.042
210 µL	1.222	1.042

[a] ^{1}H NMR titrations were carried out following the procedures detailed above.

Table S19. Changes in the ppm values of water and hydroxyl proton peaks in the ^{1}H NMR spectrum of compound **1** in DMSO-d_6 upon the addition of varying amounts of D_2O[a]

Added D_2O (μL)	H_2O and -OH
0 μL	3.314
30 μL	3.553
60 μL	3.744
90 μL	3.890
120 μL	4.003
150 μL	4.092
180 μL	4.165
210 μL	4.224

[a] ^{1}H NMR titrations were carried out following the procedures detailed above.

Summary Tables for Kinetic Studies

Table S20. Changes in the normalized integration value of bimane **1** in a 1:1 acetonitrile: water solvent system over time, with the decrease in the integration value over time[a]

Time (min)	Normalized integration
0	1.00
2	0.99
4	0.99
6	0.98
8	0.98
10	0.97
12	0.97
14	0.96
16	0.96
18	0.95
20	0.95
22	0.94
24	0.94
26	0.93
28	0.92
30	0.92
32	0.91
34	0.91
36	0.91
38	0.90
40	0.90
42	0.89
44	0.89
46	0.88
48	0.88
50	0.87

[a] Integration values were normalized to 1.00, and were calculated based on integration of the fluorescence emission from excitation at 450 nm. All integration was done vs. wavenumber on the X-axis.

SUMMARY FIGURES

*Summary Figures from Bimane **1** Synthesis*

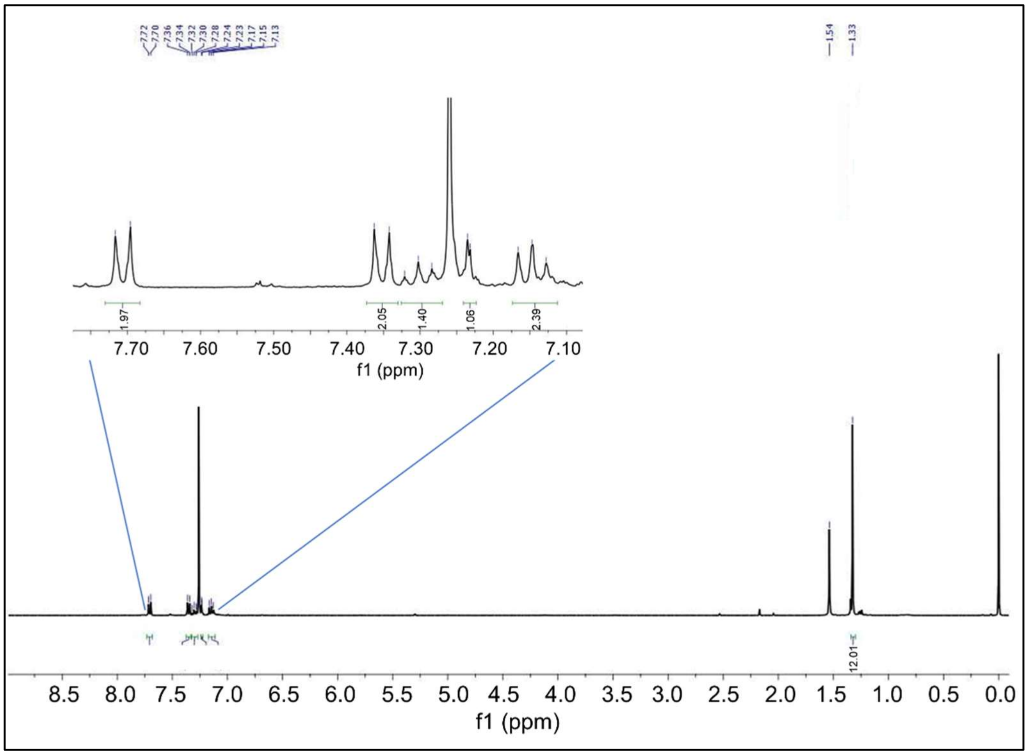

Figure S5. ^{1}H NMR spectrum of compound **1**

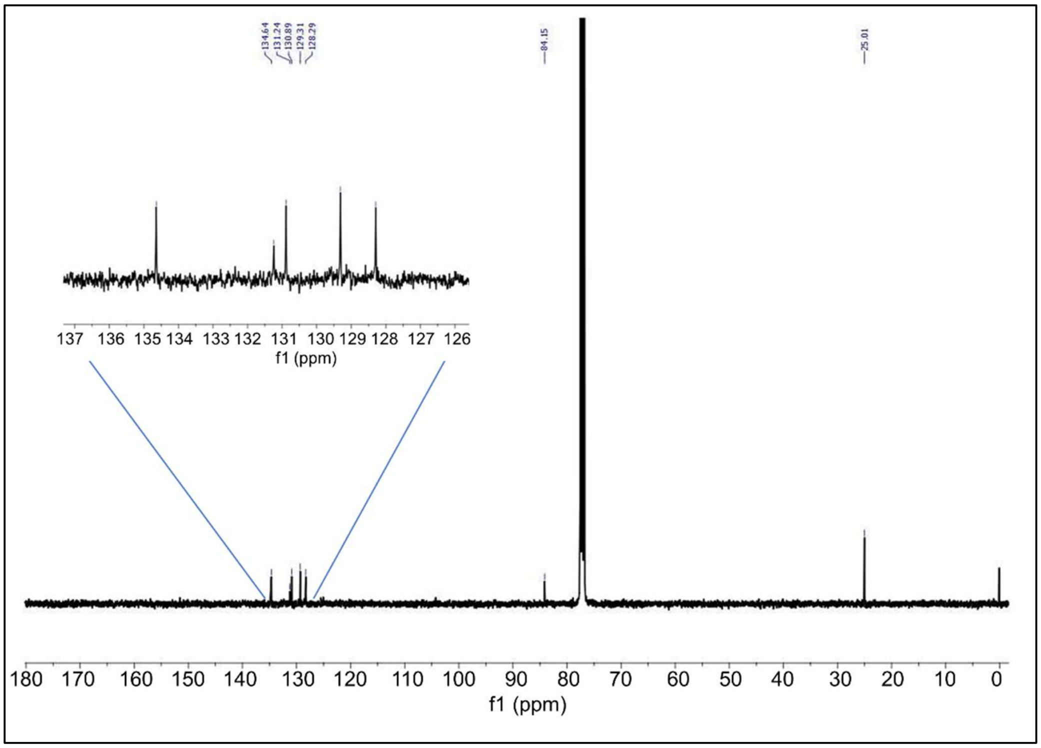

Figure S6. ^{13}C NMR spectrum of compound **1**

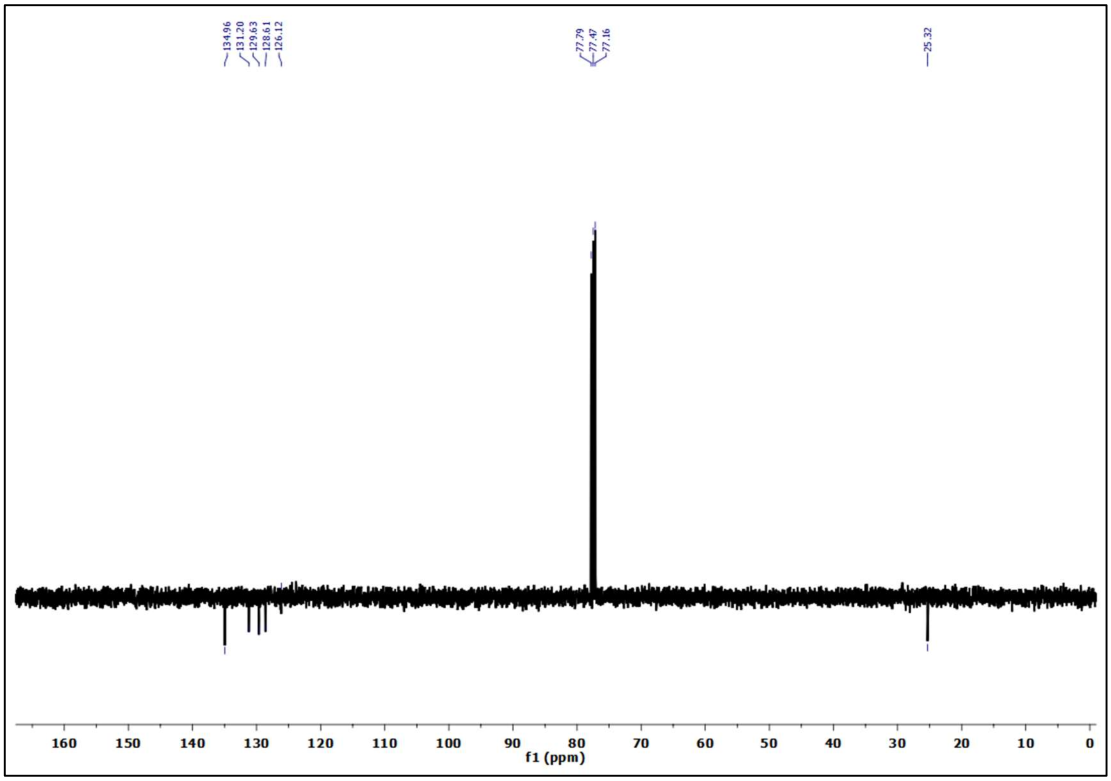

Figure S7. DEPTQ spectrum for compound **1**

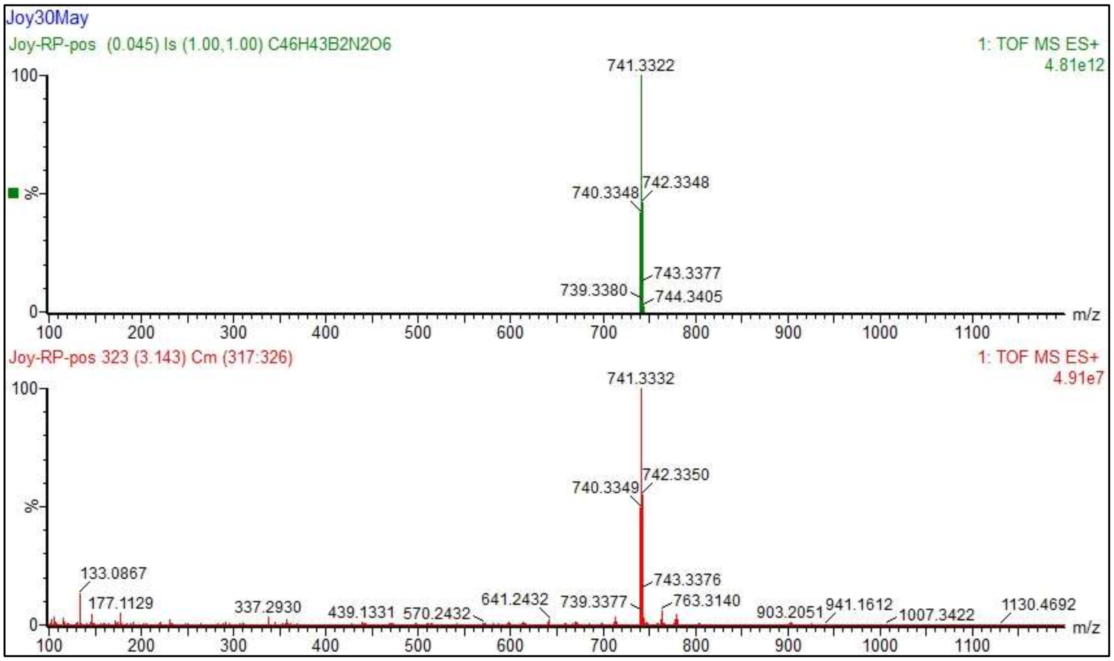

Figure S8. High resolution mass spectrum (HRMS) of compound **1** (top: predicted spectrum; bottom: experimentally obtained spectrum)

Summary Figures for UV-Visible and Fluorescence Studies

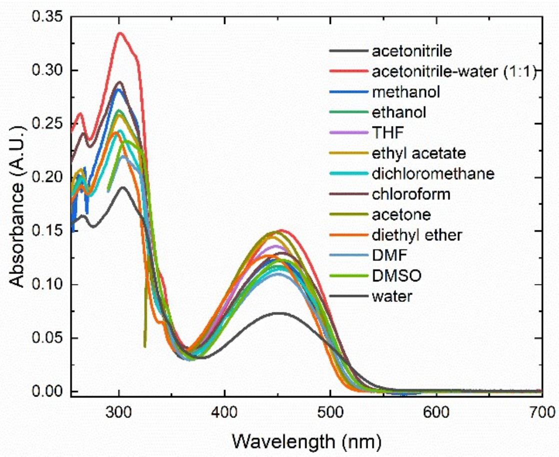

Figure S9. UV-visible absorption spectra of bimane **1** in various solvent systems ([**1**] = 10 μM)

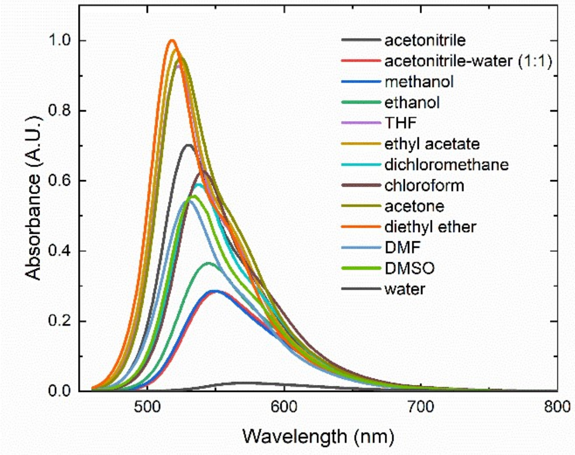

Figure S10. Normalized fluorescence emission spectra of bimane **1** in various solvent systems ([**1**] = 10 μM; λ_{ex} = 450 nm)

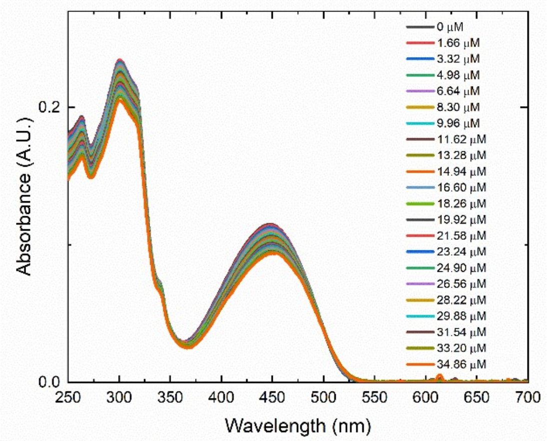

Figure S11. UV-visible absorption spectra of bimane **1** in acetonitrile in the presence of increasing concentrations of water ([**1**] = 10 μM; [H_2O] = 0 μM-34.86 μM)

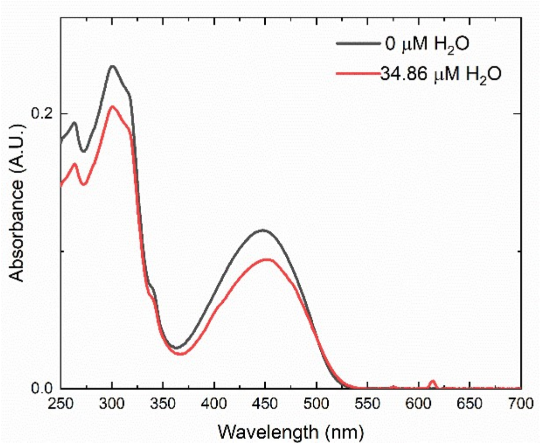

Figure S12. Summary of the UV-visible absorption spectra of bimane **1** in acetonitrile in the presence of increasing concentrations of water ([**1**] = 10 μM; [H_2O] = 0 μM-34.86 μM)

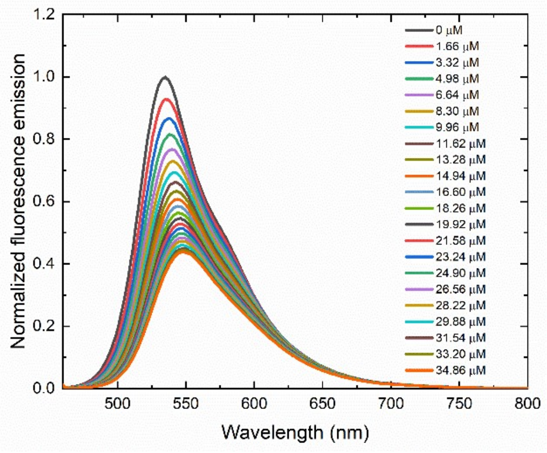

Figure S13. Normalized fluorescence emission spectra of bimane **1** in acetonitrile with increasing concentrations of water ([**1**] = 10 μM; [H_2O] = 0 μM-34.86 μM; λ_{ex} = 450 nm)

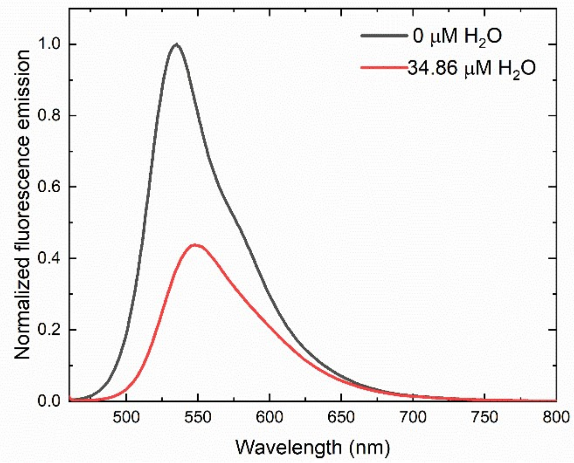

Figure S14. Summary of the normalized fluorescence emission spectra of bimane **1** in acetonitrile with increasing concentrations of water ([**1**] = 10 μM; [H_2O] = 0 μM-34.86 μM; λ_{ex} = 450 nm)

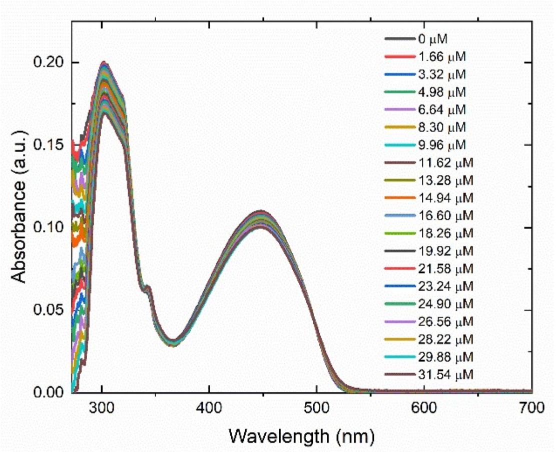

Figure S15. UV-visible absorption spectra of bimane **1** in tetrahydrofuran the presence of increasing concentrations of water ([**1**] = 10 μM; [H_2O] = 0 μM-31.54 μM)

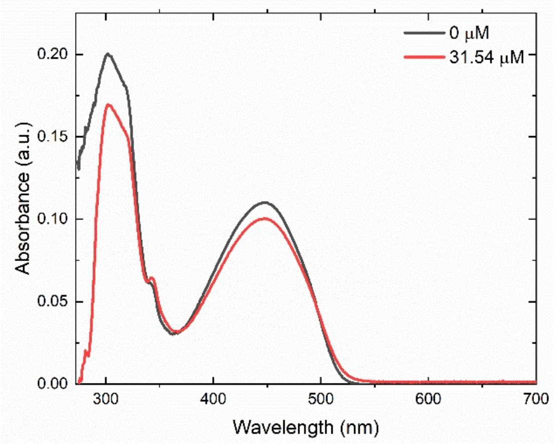

Figure S16. Summary of the UV-visible absorption spectra of bimane **1** in tetrahydrofuran in the presence of increasing concentrations of water ([**1**] = 10 μM; [H_2O] = 0 μM-31.54 μM)

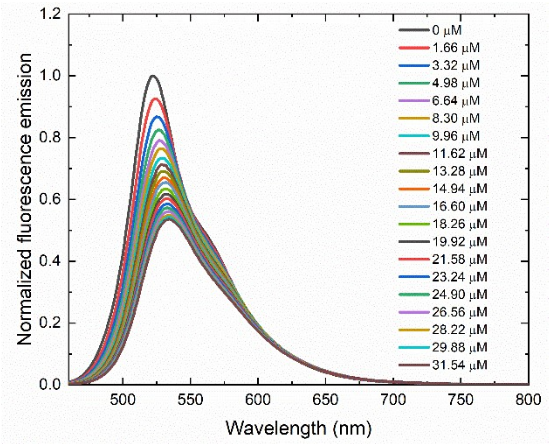

Figure S17. Normalized fluorescence emission spectra of bimane **1** in tetrahydrofuran with increasing concentrations of water ([**1**] = 10 μM; [H_2O] = 0 μM-31.54 μM; λ_{ex} = 450 nm)

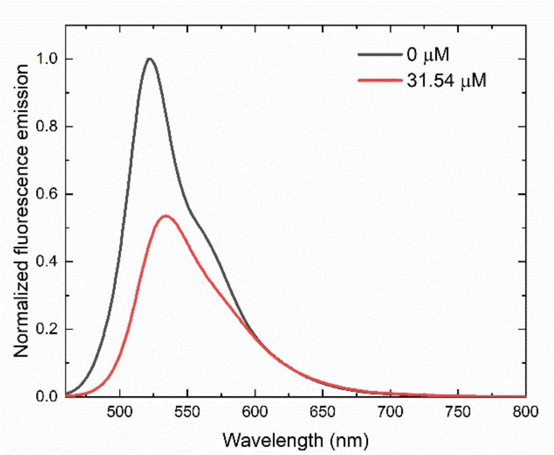

Figure S18. Summary of the normalized fluorescence emission spectra of bimane **1** in tetrahydrofuran with increasing concentrations of water ([**1**] = 10 μM; [H_2O] = 0 μM-34.86 μM; λ_{ex} = 450 nm)

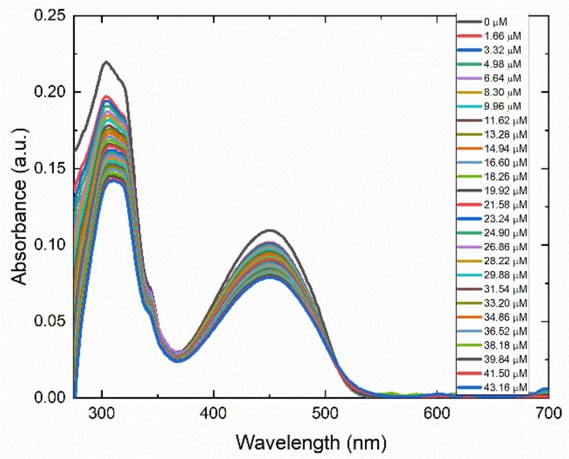

Figure S19. UV-visible absorption spectra of bimane **1** in *N,N*-dimethylformamide in the presence of increasing concentrations of water ([**1**] = 10 μM; [H_2O] = 0 μM-43.16 μM)

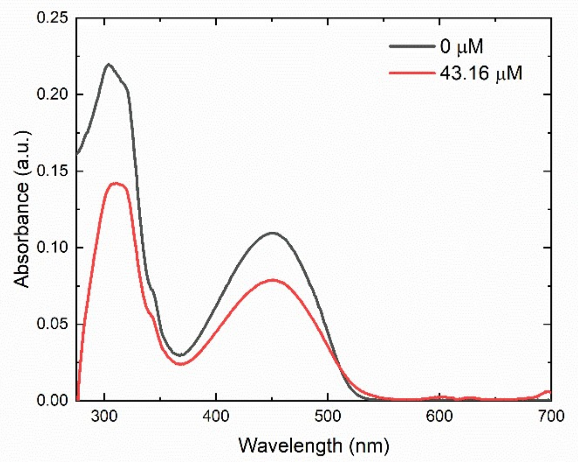

Figure S20. Summary of the UV-visible absorption spectra of bimane **1** in *N,N*-dimethylformamide in the presence of increasing concentrations of water ([**1**] = 10 μM; [H_2O] = 0 μM-43.16 μM)

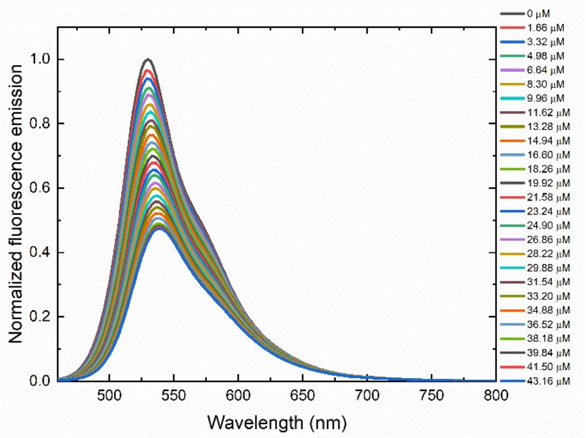

Figure S21. Normalized fluorescence emission spectra of bimane **1** in *N,N*-dimethylformamide with increasing concentrations of water ([**1**] = 10 μM; [H_2O] = 0 μM-43.16 μM; λ_{ex} = 450 nm)

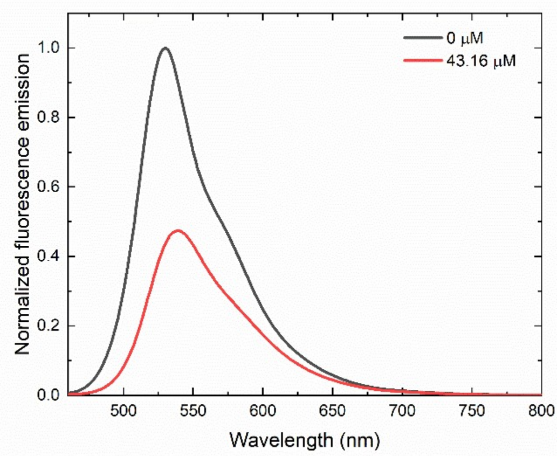

Figure S22. Summary of the normalized fluorescence emission spectra of bimane **1** in *N,N*-dimethylformamide with increasing concentrations of water ([**1**] = 10 μM; [H_2O] = 0 μM-43.16 μM; λ_{ex} = 450 nm)

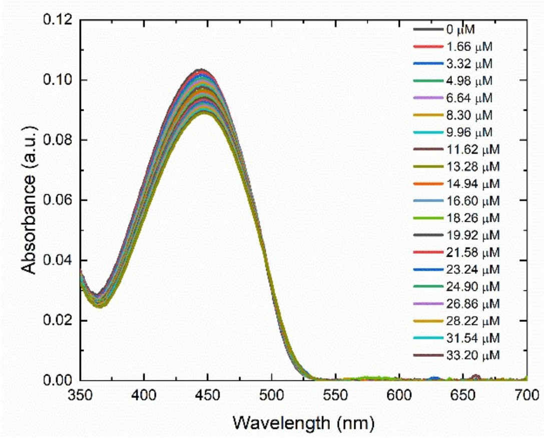

Figure S23. UV-visible absorption spectra of bimane **1** in acetone in the presence of increasing concentrations of water ([**1**] = 10 μM; [H_2O] = 0 μM-33.20 μM)

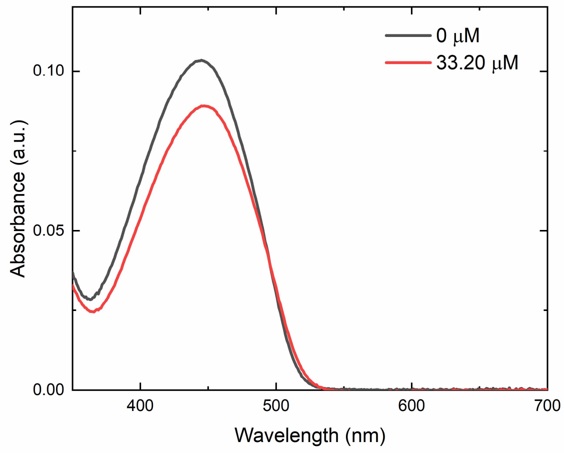

Figure S24. Summary of the UV-visible absorption spectra of bimane **1** in acetone in the presence of increasing concentrations of water ([**1**] = 10 μM; [H_2O] = 0 μM-33.20 μM)

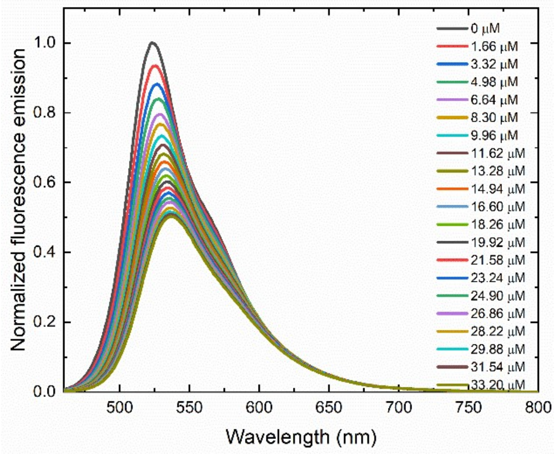

Figure S25. Normalized fluorescence emission spectra of bimane **1** in acetone with increasing concentrations of water ([**1**] = 10 μM; [H_2O] = 0 μM-33.20 μM; λ_{ex} = 450 nm)

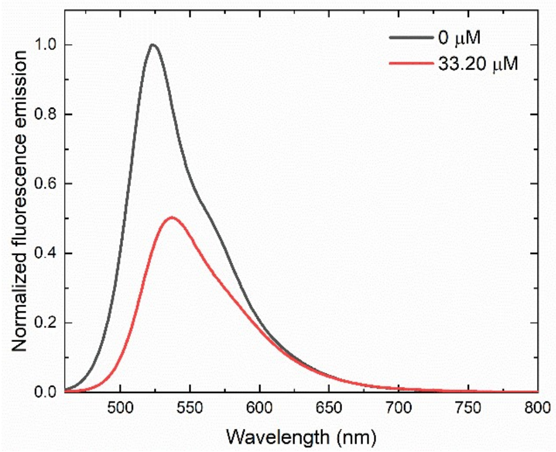

Figure S26. Summary of the normalized fluorescence emission spectra of bimane **1** in acetone with increasing concentrations of water ([**1**] = 10 μM; [H_2O] = 0 μM-33.20 μM; λ_{ex} = 450 nm)

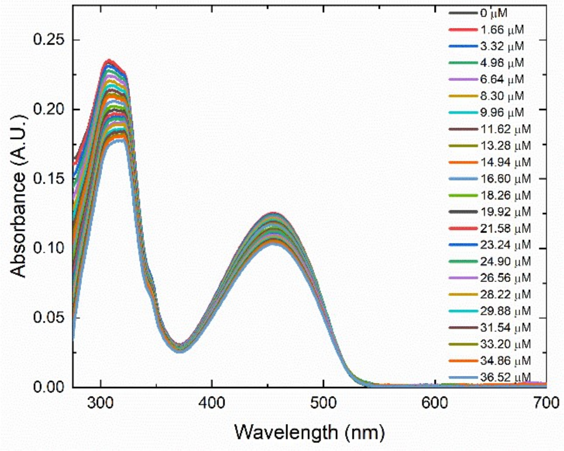

Figure S27. UV-visible absorption spectra of bimane **1** in dimethylsulfoxide (DMSO) in the presence of increasing concentrations of water ([**1**] = 10 µM; [H_2O] = 0 µM-36.52 µM)

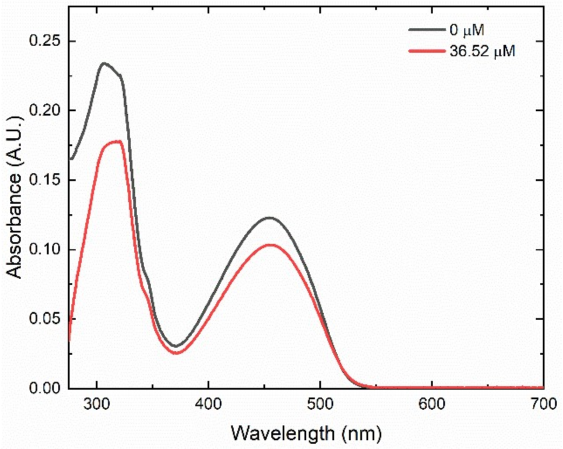

Figure S28. Summary of the UV-visible absorption spectra of bimane **1** in dimethylsulfoxide (DMSO) in the presence of increasing concentrations of water ([**1**] = 10 µM; [H_2O] = 0 µM-36.52 µM)

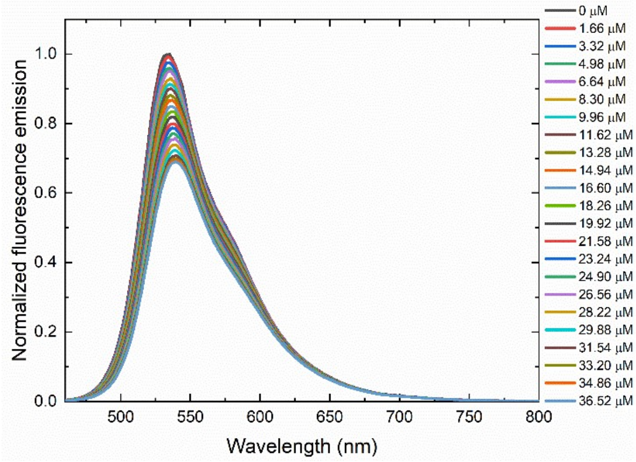

Figure S29. Normalized fluorescence emission spectra of bimane **1** in dimethylsulfoxide (DMSO) with increasing concentrations of water ([**1**] = 10 µM; [H_2O] = 0 µM-36.52 µM; λ_{ex} = 450 nm)

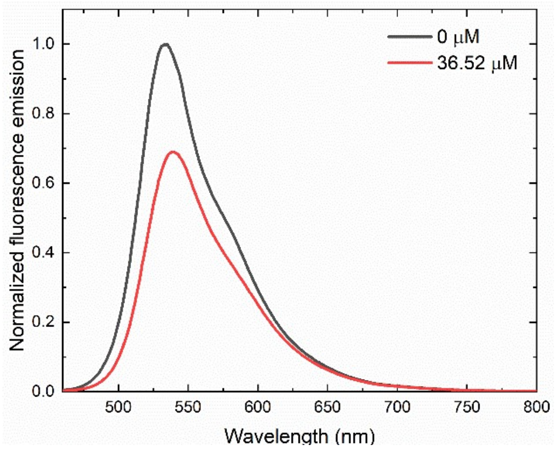

Figure S30. Summary of the normalized fluorescence emission spectra of bimane **1** in dimethylsulfoxide (DMSO) with increasing concentrations of water ([**1**] = 10 μM; [H_2O] = 0 μM-36.52 μM; λ_{ex} = 450 nm)

Summary Figures for Limit of Detection Studies

Fluorescence-Based Studies

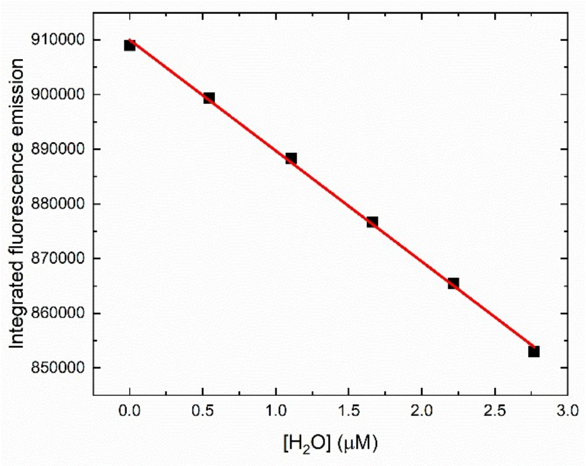

Figure S31. Calibration graph for the LOD calculations of water in DMF

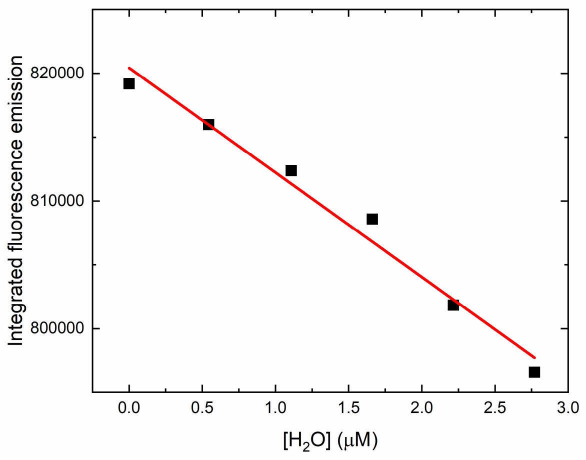

Figure S32. Calibration graph for the LOD calculations of water in DMSO

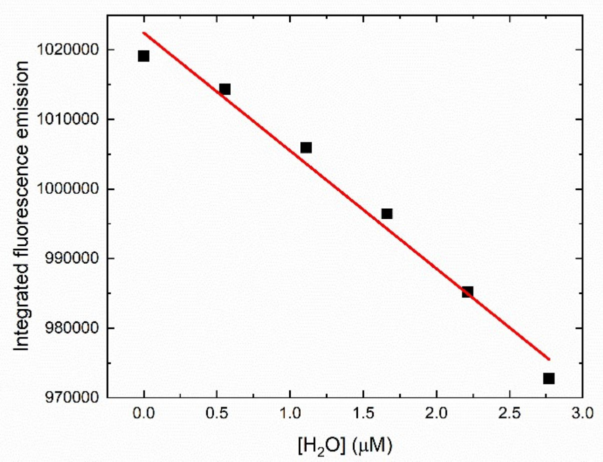

Figure S33. Calibration graph for the LOD calculations of water in acetonitrile

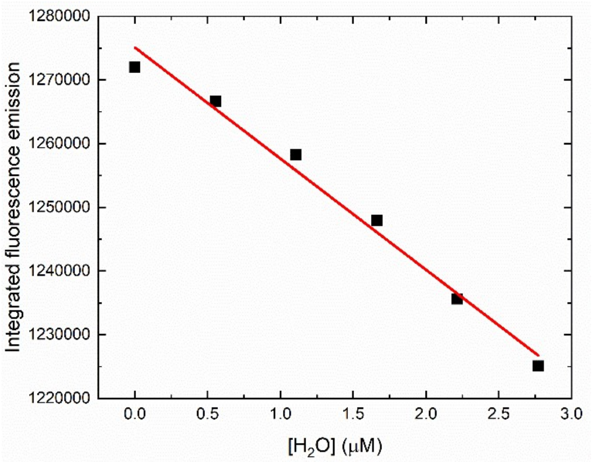

Figure S34. Calibration graph for the LOD calculations of water in THF

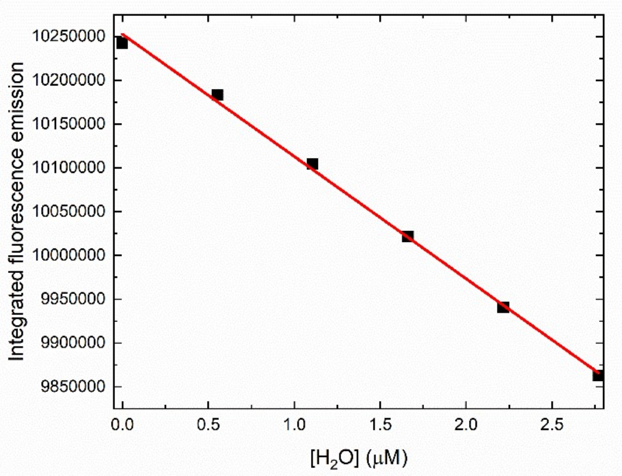

Figure S35. Calibration graph for the LOD calculations of water in acetone

Colorimetric-Based Studies

<u>ACETONITRILE</u>

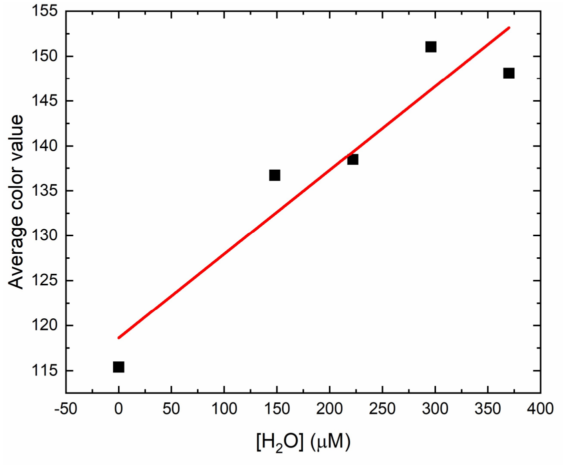

Figure S36. Calibration graph for the LOD calculations of water in acetonitrile using average red value

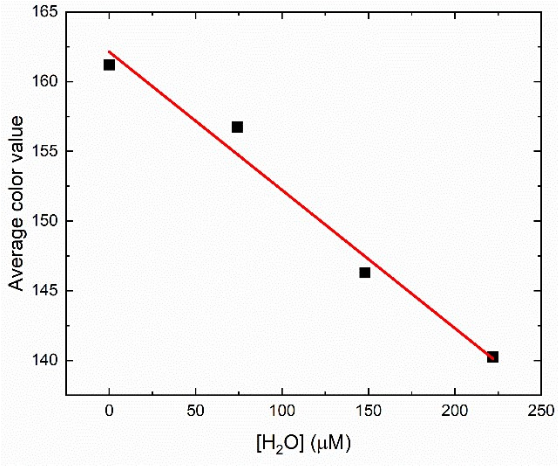

Figure S37. Calibration graph for the LOD calculations of water in acetonitrile using average green value

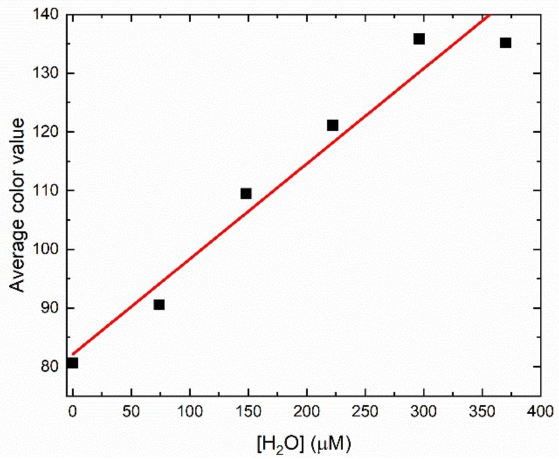

Figure S38. Calibration graph for the LOD calculations of water in acetonitrile using average blue value

ACETONE

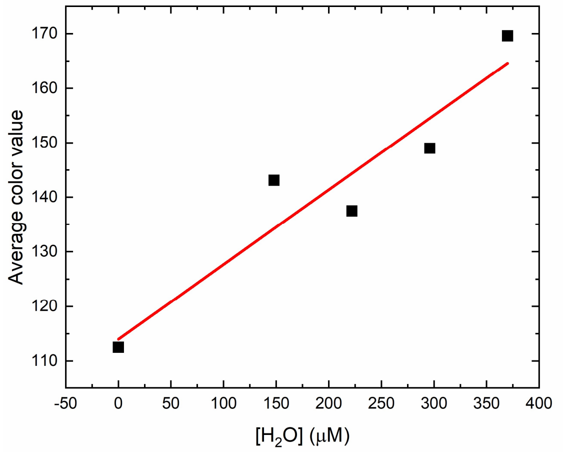

Figure S39. Calibration graph for the LOD calculations of water in acetone using average red value

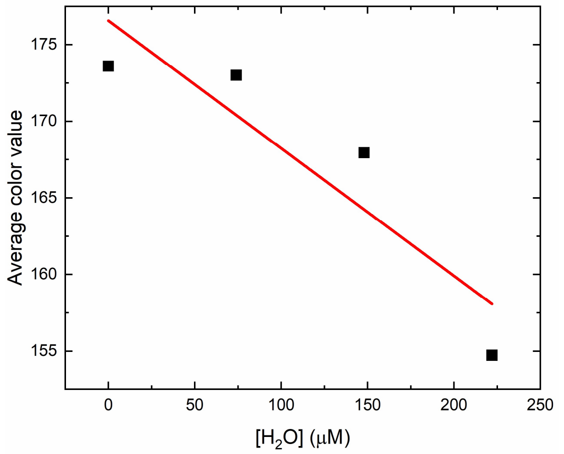

Figure S40. Calibration graph for the LOD calculations of water in acetone using average green value

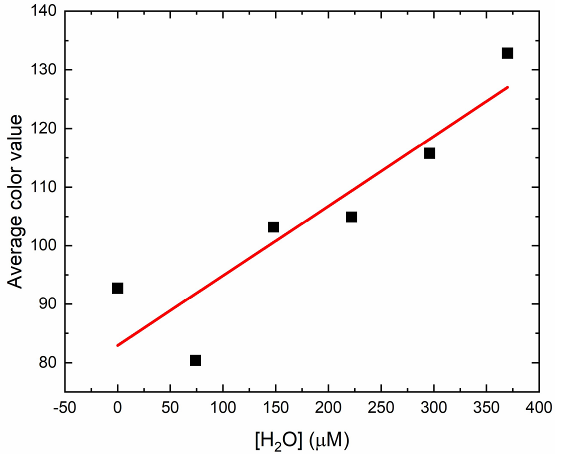

Figure S41. Calibration graph for the LOD calculations of water in acetone using average blue value

DMF

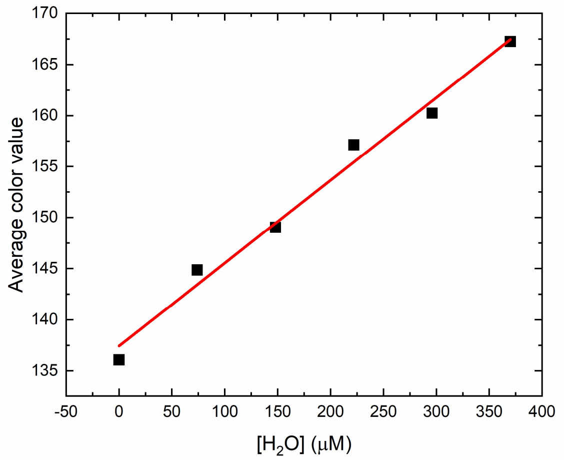

Figure S42. Calibration graph for the LOD calculations of water in DMF using average red value

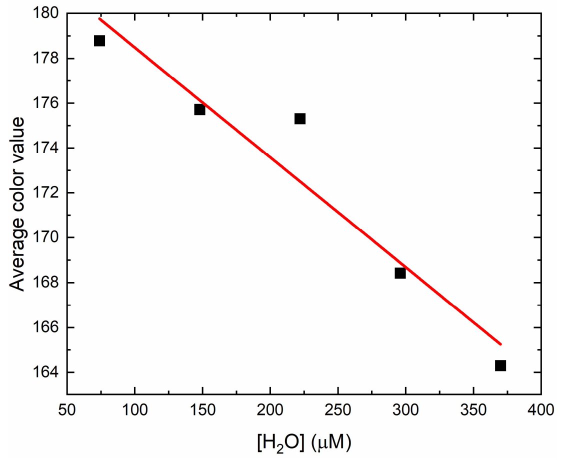

Figure S43. Calibration graph for the LOD calculations of water in DMF using average green value

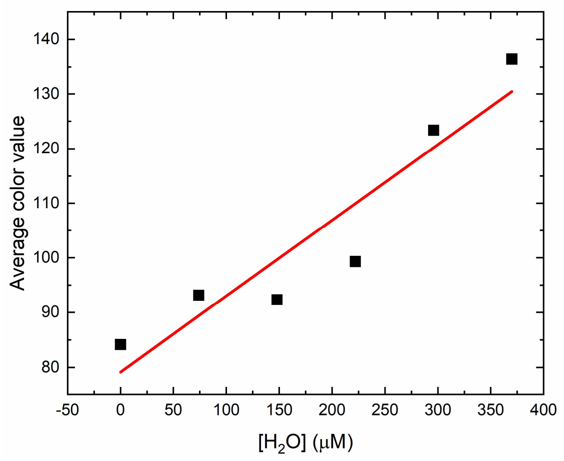

Figure S44. Calibration graph for the LOD calculations of water in DMF using average blue value

THF

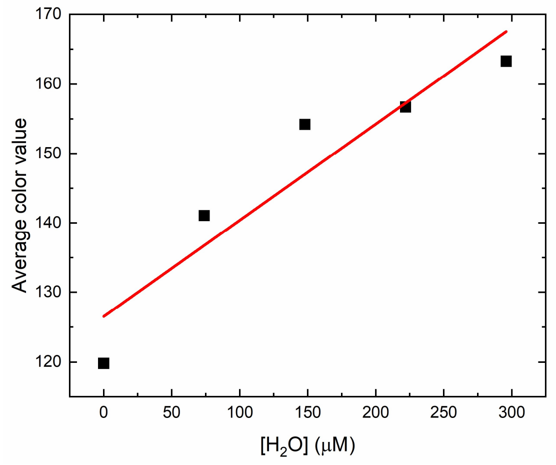

Figure S45. Calibration graph for the LOD calculations of water in THF using average red value

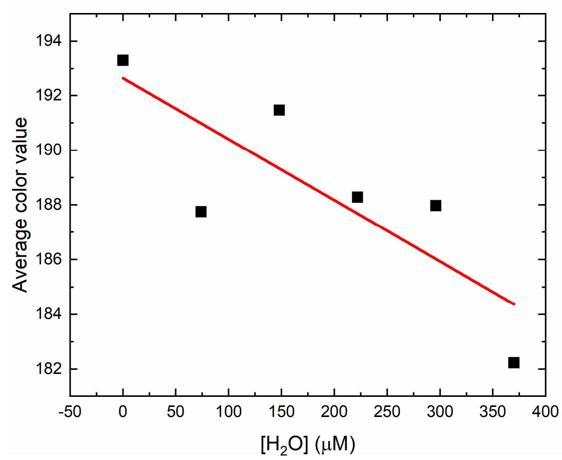

Figure S46. Calibration graph for the LOD calculations of water in THF using average green value

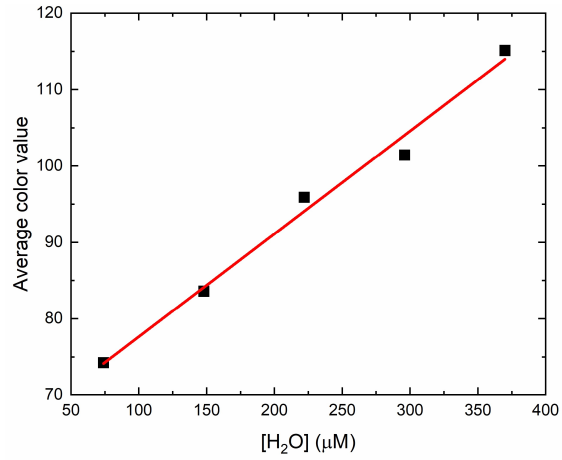

Figure S47. Calibration graph for the LOD calculations of water in THF using average blue value

DMSO

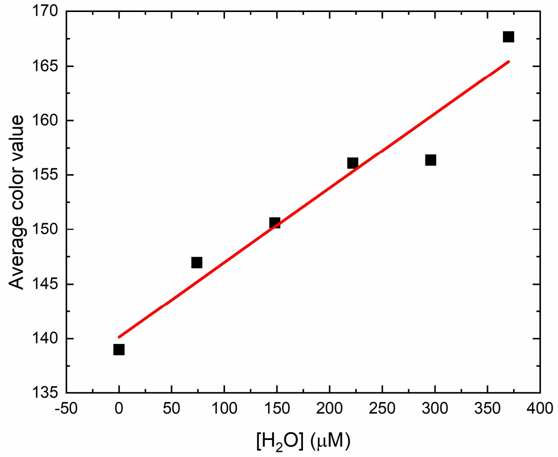

Figure S48. Calibration graph for the LOD calculations of water in DMSO using average red value

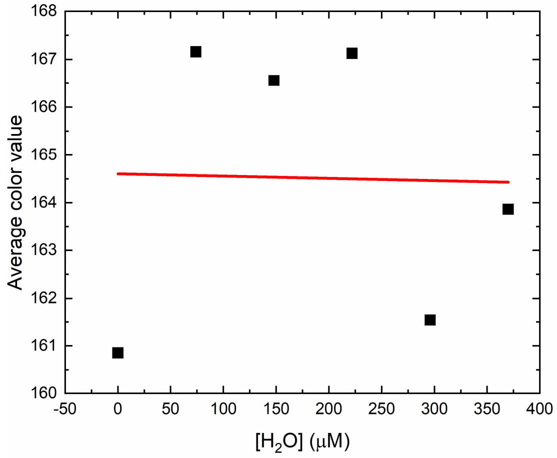

Figure S49. Calibration graph for the LOD calculations of water in DMSO using average green value

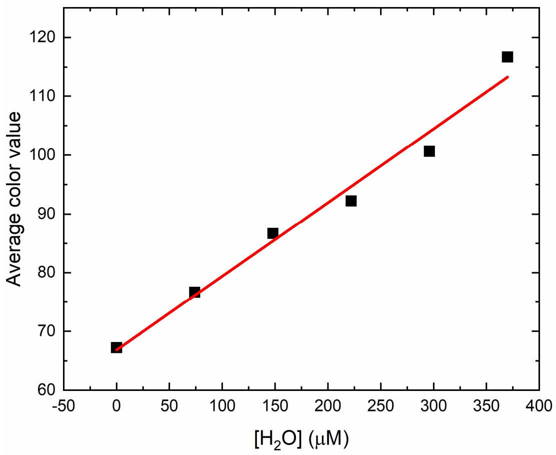

Figure S50. Calibration graph for the LOD calculations of water in DMSO using average blue value

Colorimetric photos used for LOD calculations

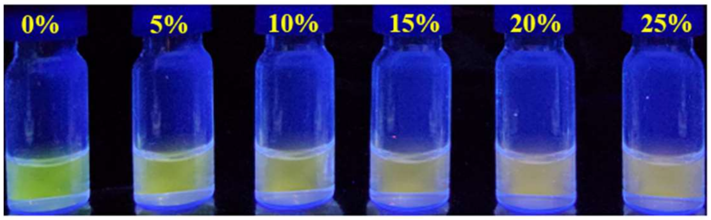

Figure S51. A photograph of the solutions of bimane **1** in acetonitrile with increasing percentages of water, under irradiation with a long-wave, hand-held TLC lamp (365 nm) (L-R: 0%, 5%, 10%, 15%, 20%, 25%)

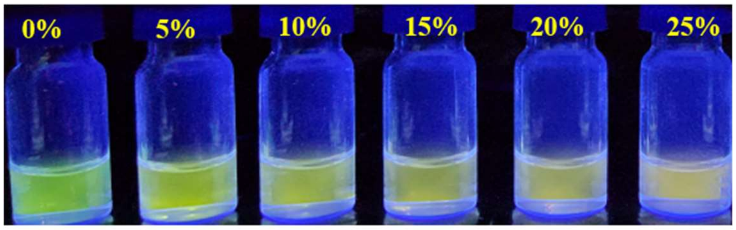

Figure S52. A photograph of the solutions of bimane **1** in acetone with increasing percentages of water, under irradiation with a long-wave, hand-held TLC lamp (365 nm) (L-R: 0%, 5%, 10%, 15%, 20%, 25%)

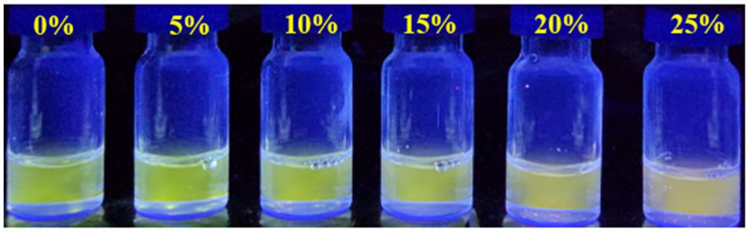

Figure S53. A photograph of the solutions of bimane **1** in DMF with increasing percentages of water, under irradiation with a long-wave, hand-held TLC lamp (365 nm) (L-R: 0%, 5%, 10%, 15%, 20%, 25%)

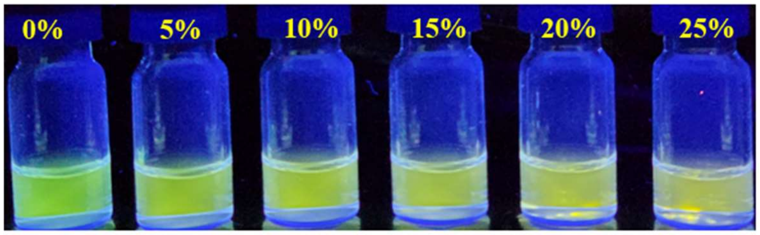

Figure S54. A photograph of the solutions of bimane **1** in THF with increasing percentages of water, under irradiation with a long-wave, hand-held TLC lamp (365 nm) (L-R: 0%, 5%, 10%, 15%, 20%, 25%)

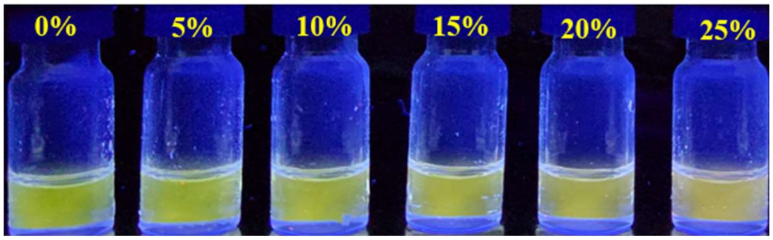

Figure S55. A photograph of the solutions of bimane **1** in DMSO with increasing percentages of water, under irradiation with a long-wave, hand-held TLC lamp (365 nm) (L-R: 0%, 5%, 10%, 15%, 20%, 25%)

Summary Figures for Solution State Studies

Solvent specific changes in the solution color under ambient and long wave UV light (365 nm)

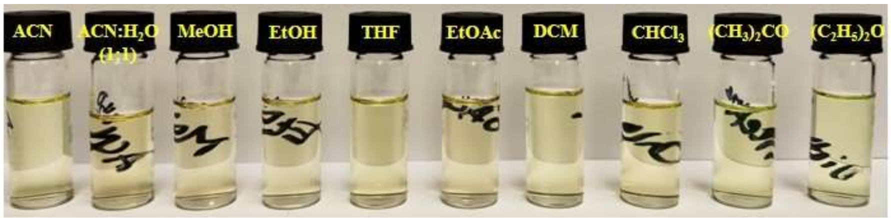

Figure S56. A photograph of bimane **1** in various solvent systems under ambient lighting conditions ([**1**] = 10 µM; Left to right: acetonitrile, acetonitrile: water (1:1 vol: vol); methanol, ethanol, tetrahydrofuran, ethyl acetate, dichloromethane, chloroform, acetone, diethyl ether)

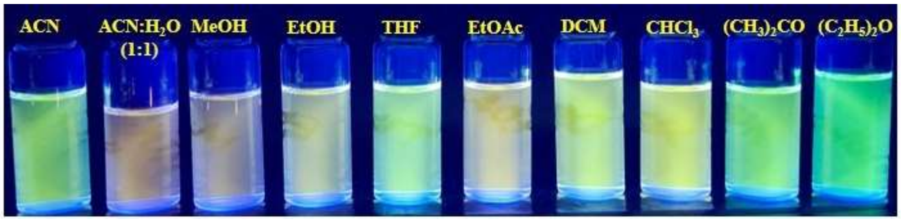

Figure S57. A photograph of bimane **1** in various solvent systems under excitation by a long-wave hand-held TLC lamp at 365 nm ([**1**] = 10 µM; Left to right: acetonitrile, acetonitrile: water (1:1 vol: vol); methanol, ethanol, tetrahydrofuran, ethyl acetate, dichloromethane, chloroform, acetone, diethyl ether)

Colorimetric changes of the bimane solution in response to added water under ambient and long-wave UV light (365 nm)

ACETONITRILE:

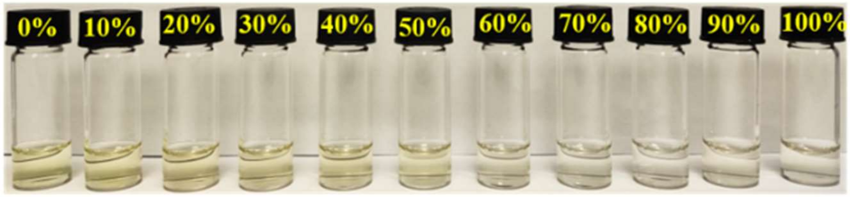

Figure S58. A photograph of bimane **1** in acetonitrile with increasing percentages of water (0-100%) under ambient lighting conditions ([**1**] = 10 μM)

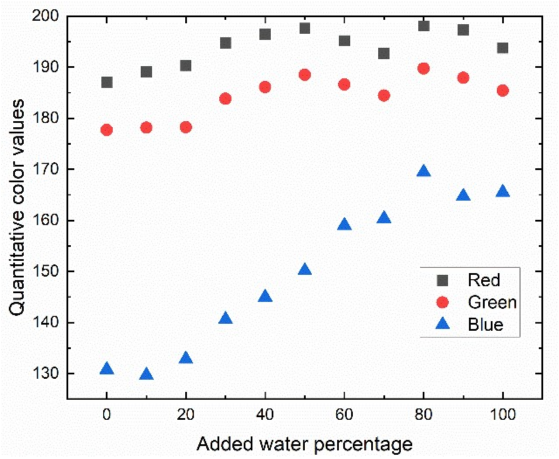

Figure S59. Quantitative color values of the solutions of bimane **1** in acetonitrile under ambient light with increasing amounts of water ([**1**] = 10 μM)

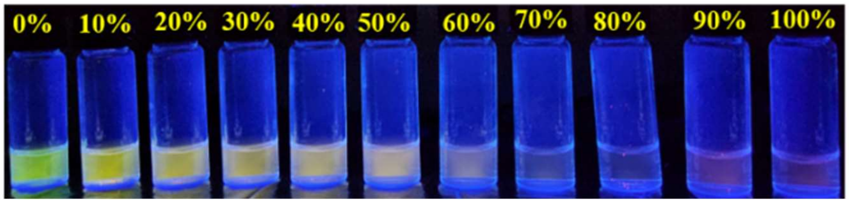

Figure S60. A photograph of bimane **1** in acetonitrile with increasing percentages of water (0-100%) with excitation by a long-wave hand-held TLC lamp at 365 nm ([**1**] = 10 μM)

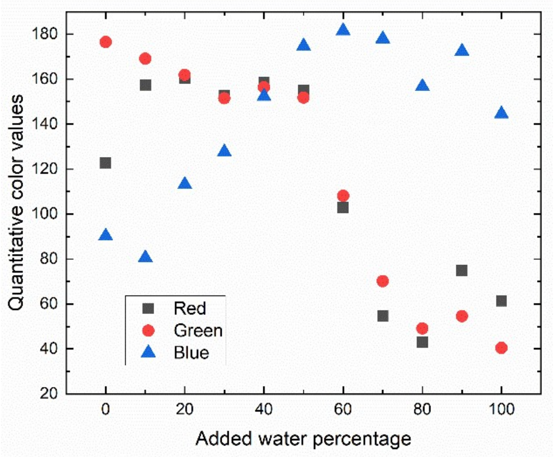

Figure S61. Quantitative color values of the solutions of bimane **1** in acetonitrile under long-wave UV light (365 nm) with increasing amounts of water ([**1**] = 10 μM)

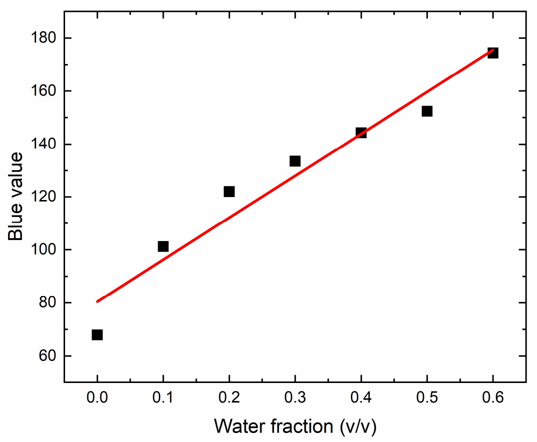

Figure S62. Changes in the average blue value of the solution of bimane **1** in acetonitrile under 365 nm irradiation as a function of increasing percentages of water, calculated using a random sampling of >10 data points in Microsoft Paint

THF:

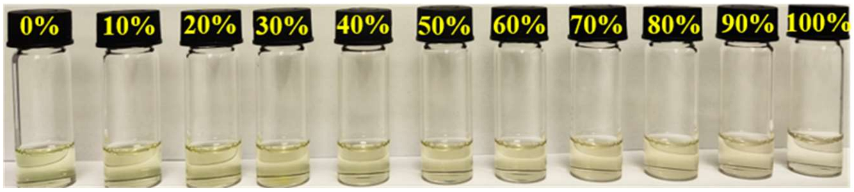

Figure S63. A photograph of bimane **1** in tetrahydrofuran with increasing percentages of water (0-100%) under ambient lighting conditions ([**1**] = 10 µM)

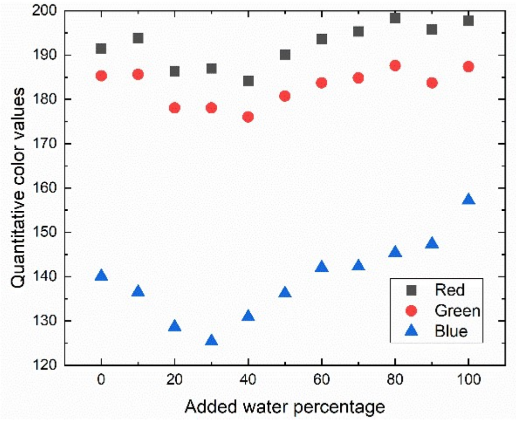

Figure S64. Quantitative color values of the solutions of bimane **1** in tetrahydrofuran under ambient light with increasing amounts of water ([**1**] = 10 µM)

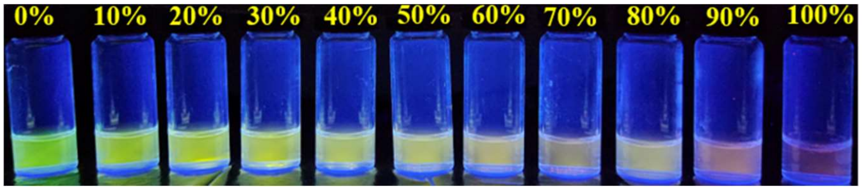

Figure S65. A photograph of bimane **1** in tetrahydrofuran with increasing percentages of water (0-100%) with excitation by a long-wave hand-held TLC lamp at 365 nm ([**1**] = 10 µM)

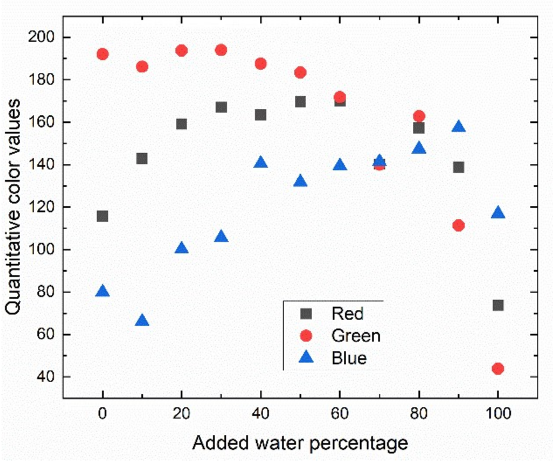

Figure S66. Quantitative color values of the solutions of bimane **1** in tetrahydrofuran under long-wave UV light (365 nm) with increasing amounts of water ([**1**] = 10 µM)

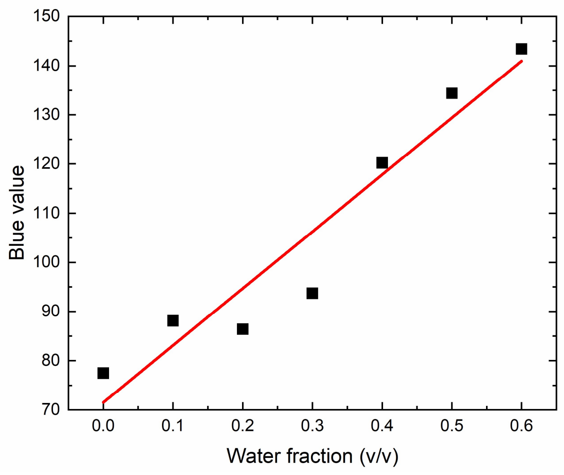

Figure S67. Changes in the average blue value of the solution of bimane **1** in THF under 365 nm irradiation as a function of increasing percentages of water, calculated using a random sampling of >10 data points in Microsoft Paint

ACETONE:

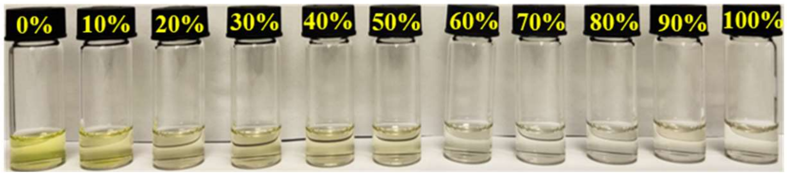

Figure S68. A photograph of bimane **1** in acetone with increasing percentages of water (0-100%) under ambient lighting conditions ([**1**] = 10 μM)

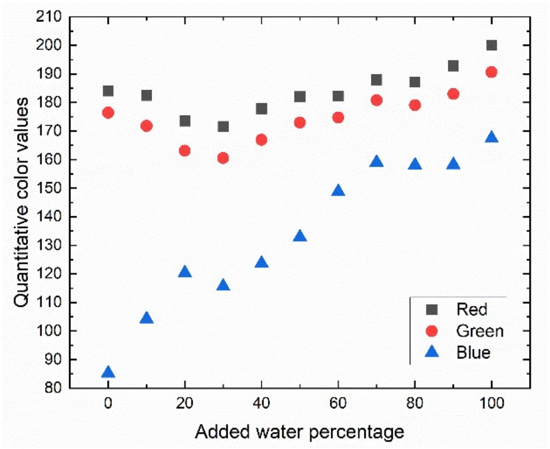

Figure S69. Quantitative color values of the solutions of bimane **1** in acetone under ambient light with increasing amounts of water ([**1**] = 10 μM)

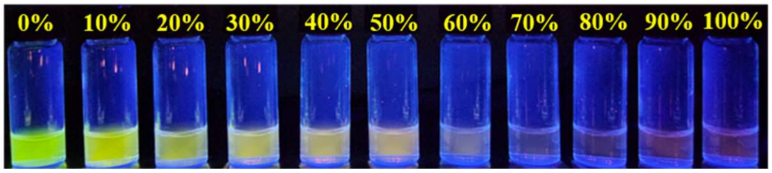

Figure S70. A photograph of bimane **1** in acetone with increasing percentages of water (0-100%) with excitation by a long-wave hand-held TLC lamp at 365 nm ([**1**] = 10 μM)

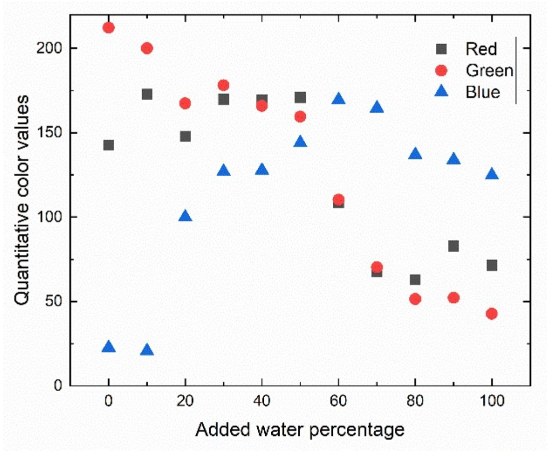

Figure S71. Quantitative color values of the solutions of bimane **1** in acetone under long-wave UV light (365 nm) with increasing amounts of water ([**1**] = 10 μM)

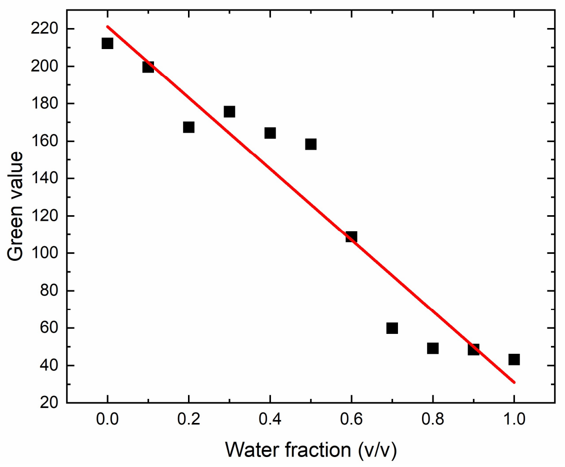

Figure S72. Changes in the average green value of the solution of bimane **1** in acetone under 365 nm irradiation as a function of increasing percentages of water, calculated using a random sampling of greater than 10 randomly selected data points in Microsoft Paint

N, N-DIMETHYLFORMAMIDE (DMF)

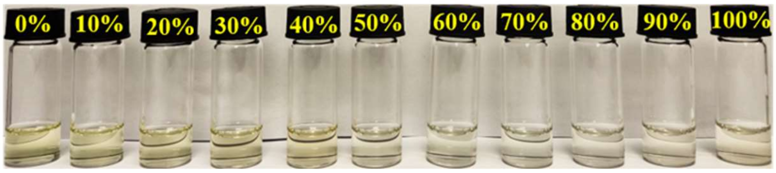

Figure S73. A photograph of bimane **1** in *N,N*-dimethylformamide with increasing percentages of water (0-100%) under ambient lighting conditions ([**1**] = 10 μM)

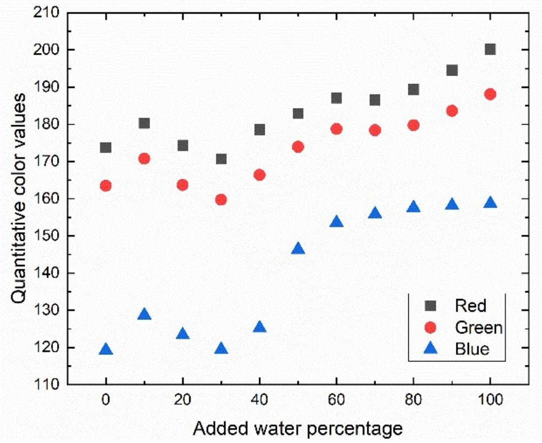

Figure S74. Quantitative color values of the solutions of bimane **1** in DMF under ambient light with increasing amounts of water ([**1**] = 10 μM)

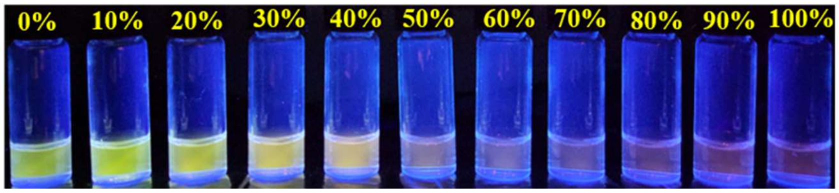

Figure S75. A photograph of bimane **1** in *N,N*-dimethylformamide with increasing percentages of water (0-100%) with excitation by a long-wave hand-held TLC lamp at 365 nm ([**1**] = 10 μM)

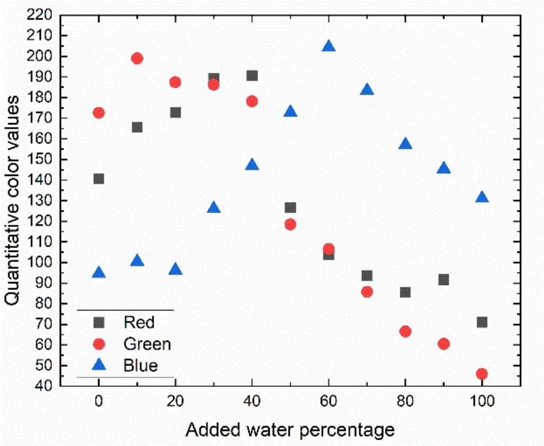

Figure S76. Quantitative color values of the solutions of bimane **1** in DMF under long-wave UV light (365 nm) with increasing amounts of water ([**1**] = 10 μM)

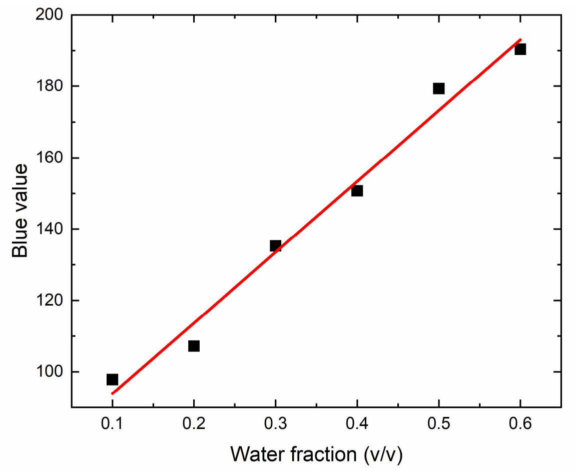

Figure S77. Changes in the average blue value of the solution of bimane **1** in DMF under 365 nm irradiation as a function of increasing percentages of water

DIMETHYLSULFOXIDE (DMSO)

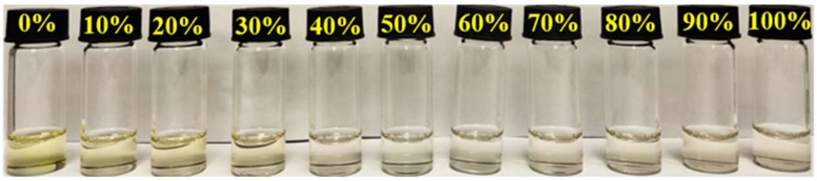

Figure S78. A photograph of bimane **1** in dimethylsulfoxide with increasing percentages of water (0-100%) under ambient lighting conditions ([**1**] = 10 μM)

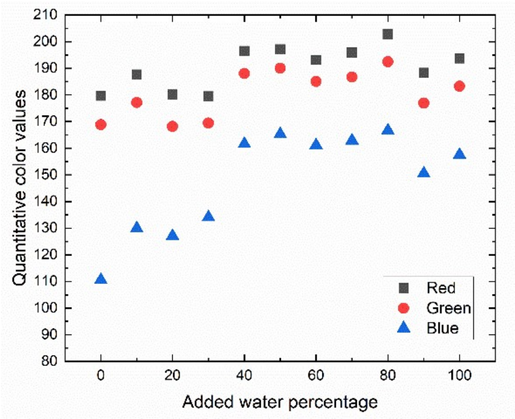

Figure S79. Quantitative color values of the solutions of bimane **1** in DMSO under ambient light with increasing amounts of water ([**1**] = 10 μM)

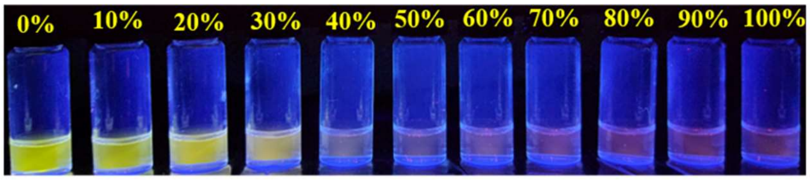

Figure S80. A photograph of bimane **1** in dimethylsulfoxide with increasing percentages of water (0-100%) with excitation by a long-wave hand-held TLC lamp at 365 nm ([**1**] = 10 μM)

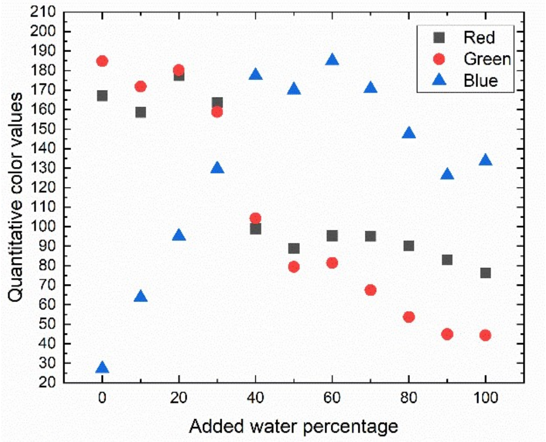

Figure S81. Quantitative color values of the solutions of bimane **1** in DMSO under long-wave UV light (365 nm) with increasing amounts of water ([**1**] = 10 μM)

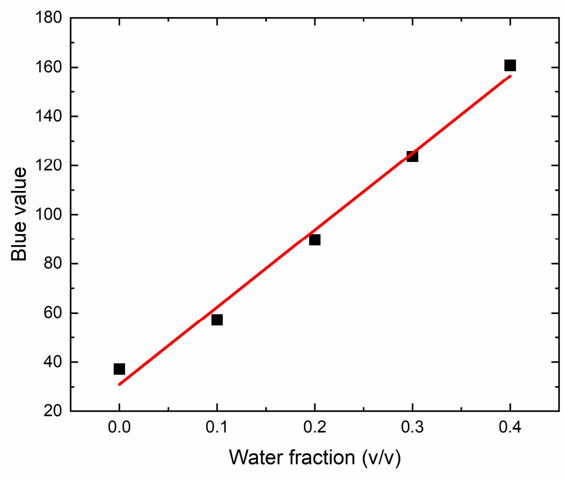

Figure S82. Changes in the average blue value of the solution of bimane **1** in DMSO under 365 nm irradiation as a function of increasing percentages of water, calculated using a random sampling of greater than 10 randomly selected data points in Microsoft Paint

Summary Figures for Solid-State Studies

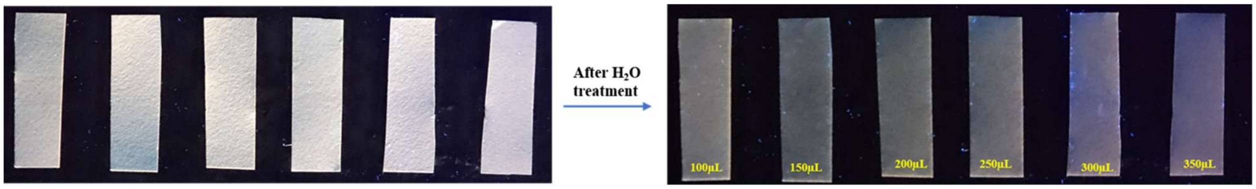

Figure S83. Changes in the appearance of papers functionalized with bimane **1** after exposure to varying quantities of water (left-to-right: 100 µL, 150 µL, 200 µL, 250 µL, 300 µL, 350 µL)

Summary Figures for ^{1}H NMR Titration Studies

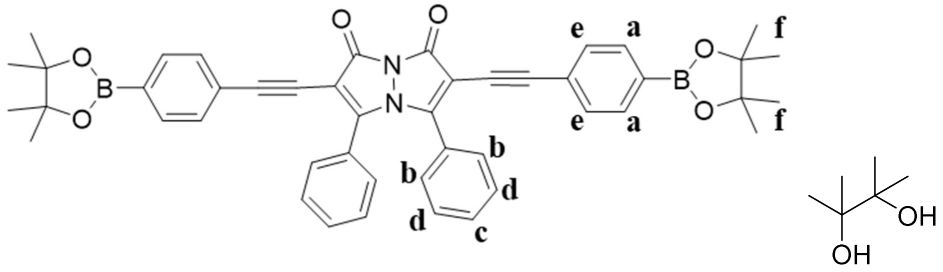

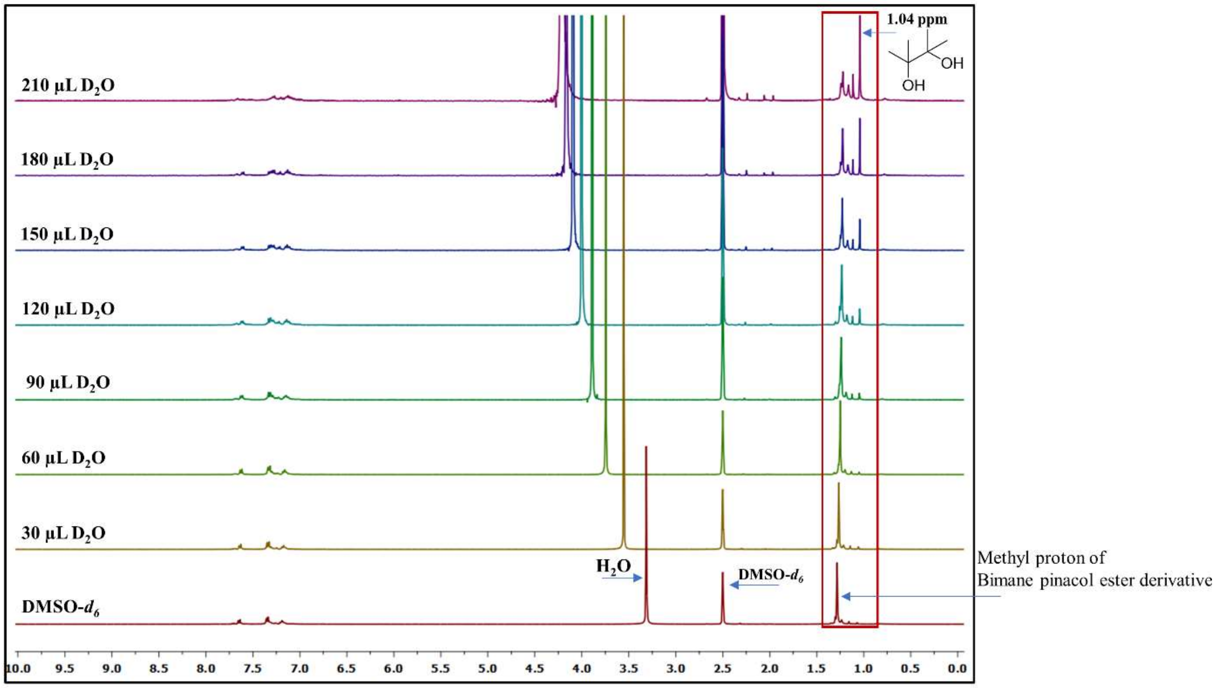

Figure S84. ^{1}H NMR spectra of compound **1** in DMSO-d_6 with increasing concentrations of D_2O, showing a decrease in the methyl peak corresponding to the bimane pinacol ester and an increase in the peak corresponding to free pinacol; bottom-to-top: 0 µL D_2O, 30 µL D_2O, 60 µL D_2O, 90 µL D_2O, 120 µL D_2O, 150 µL D_2O, 180 µL D_2O, 210 µL D_2O

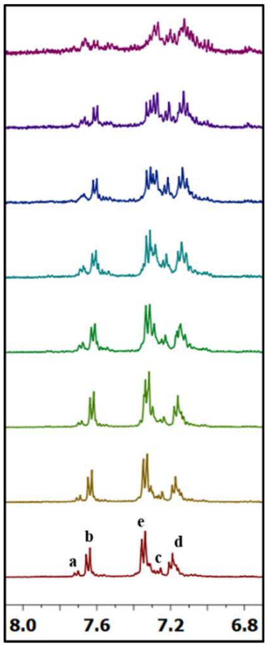

Figure S85. ^{1}H NMR spectra of compound **1** in DMSO-d_6 with increasing concentrations of D_2O, expanded aromatic region of the spectra, which shows subtle changes in the aromatic peak structure with sequential D_2O addition; bottom-to-top: 0 μL D_2O, 30 μL D_2O, 60 μL D_2O, 90 μL D_2O, 120 μL D_2O, 150 μL D_2O, 180 μL D_2O, 210 μL D_2O

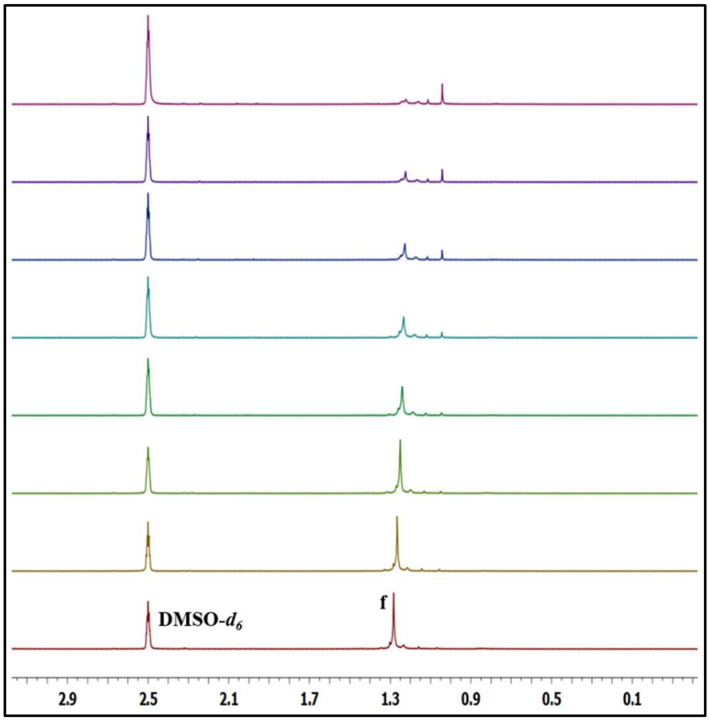

Figure S86. ^{1}H NMR spectra of compound **1** in DMSO-d_6 with increasing concentrations of D_2O, expanded aliphatic region of the spectra, which shows significant changes in the spectra with sequential D_2O addition; bottom-to-top: 0 μL D_2O, 30 μL D_2O, 60 μL D_2O, 90 μL D_2O, 120 μL D_2O, 150 μL D_2O, 180 μL D_2O, 210 μL D_2O

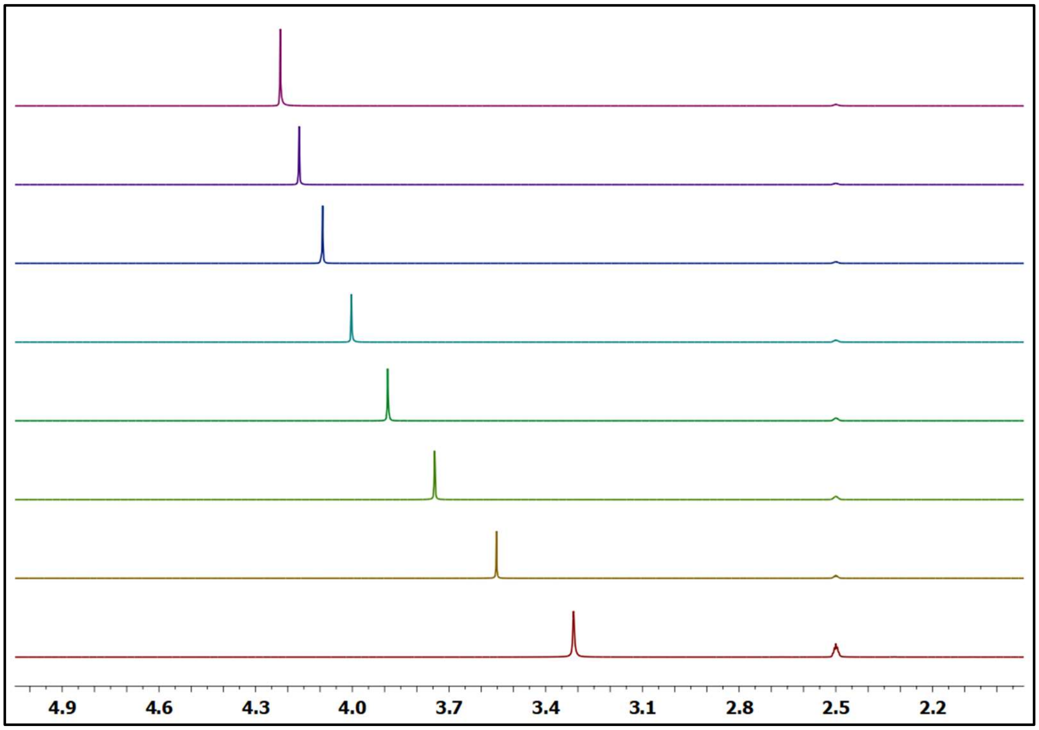

Figure S87. ^{1}H NMR spectra of compound **1** in DMSO-d_6 with increasing concentrations of D_2O, expanded hydroxyl and water peaks' region of the spectra, which shows significant changes in the spectra with sequential D_2O addition; bottom-to-top: 0 µL D_2O, 30 µL D_2O, 60 µL D_2O, 90 µL D_2O, 120 µL D_2O, 150 µL D_2O, 180 µL D_2O, 210 µL D_2O

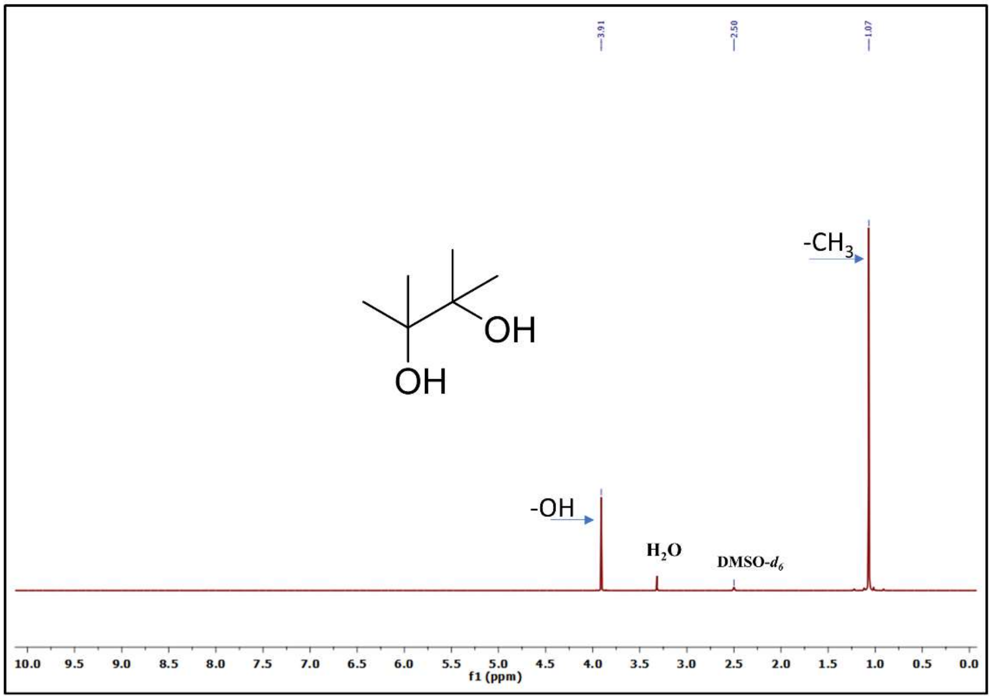

Figure S88. A ^{1}H NMR spectrum of pinacol, acquired for comparison with the spectra acquired during the ^{1}H NMR titration investigations

Summary Figures for High Resolution Mass Spectrometry Studies

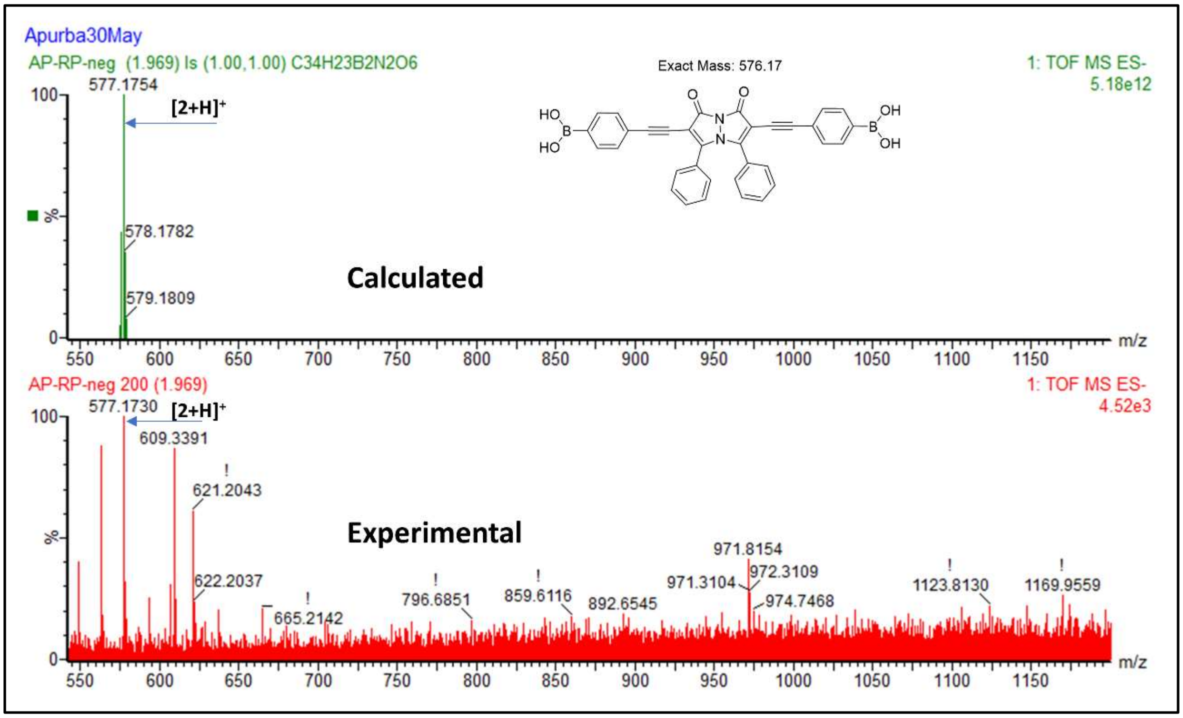

Figure S89. High resolution mass spectrum of compound **1** in a 1:1 water: acetonitrile mixture, showing a mass signal that corresponds to the mass of the hydrolyzed boronic acid (top: calculated; bottom: observed)

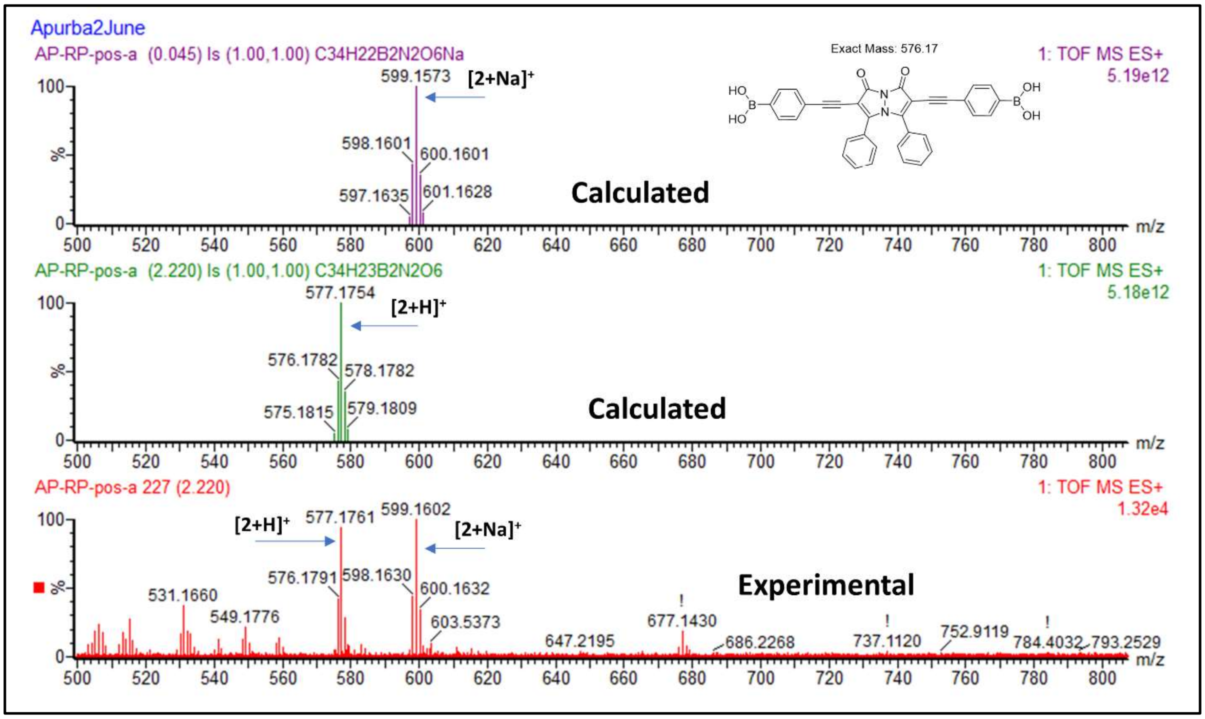

Figure S90. High resolution mass spectrum of compound **1** in a 1:1 methanol solvent, showing a mass signal that corresponds to the mass of the hydrolyzed boronic acid (top: calculated with sodium cations; middle: calculated with protons; bottom: observed)

Summary Figures for Kinetic Studies

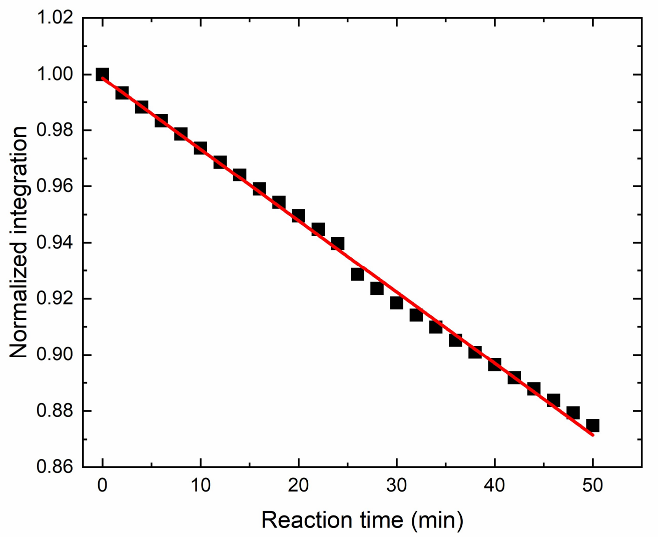

Figure S91. An illustration of the linear relationship between the normalized integration of bimane **1** in an acetonitrile-water solvent system (1:1 *vol: vol*; [**1**] = 10 μM) and the time (in minutes) allowed for the solution.

Summary Figures for Solid-State Reversibility Studies

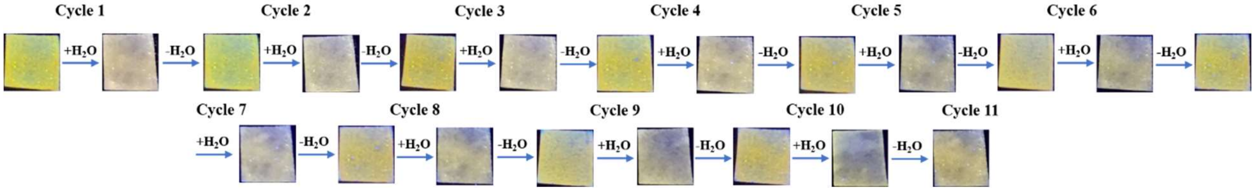

Figure S95. Photograph of the bimane **1**-functionalized paper with 11 cycles of water exposure followed by drying

Humidity Sensor Reversibility

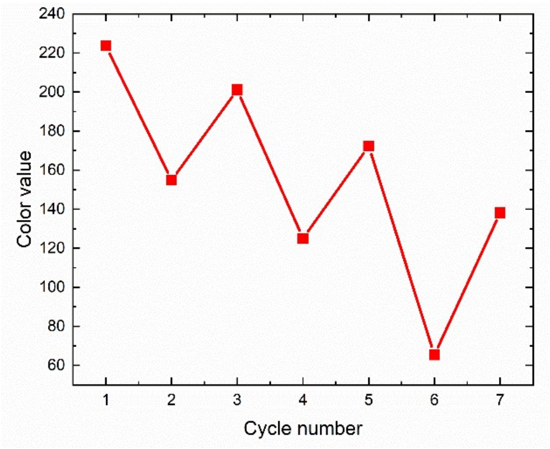

Figure S96. Illustration of the switching of the red value of the bimane-functionalized paper with 7 cycles of exposure to a high humidity atmosphere (99.9% relative humidity) and an ambient atmosphere (54% relative humidity)

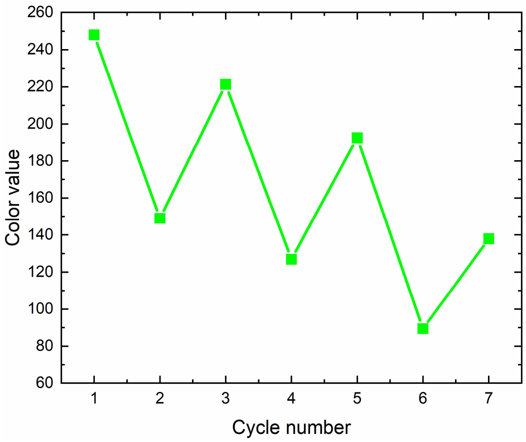

Figure S97. Illustration of the switching of the green value of the bimane-functionalized paper with 7 cycles of exposure to a high humidity atmosphere (99.9% relative humidity) and an ambient atmosphere (54% relative humidity)

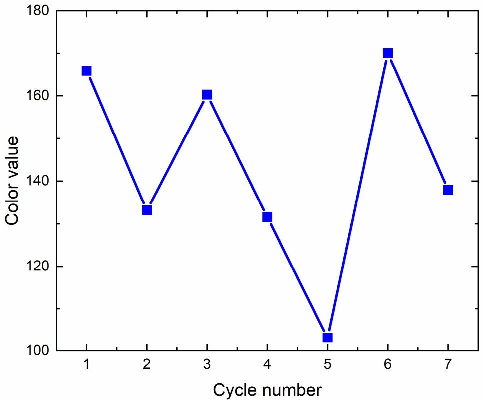

Figure S98. Illustration of the switching of the blue value of the bimane-functionalized paper with 7 cycles of exposure to a high humidity atmosphere (99.9% relative humidity) and an ambient atmosphere (54% relative humidity)

Summary Figures for Quantum Yield Experiments

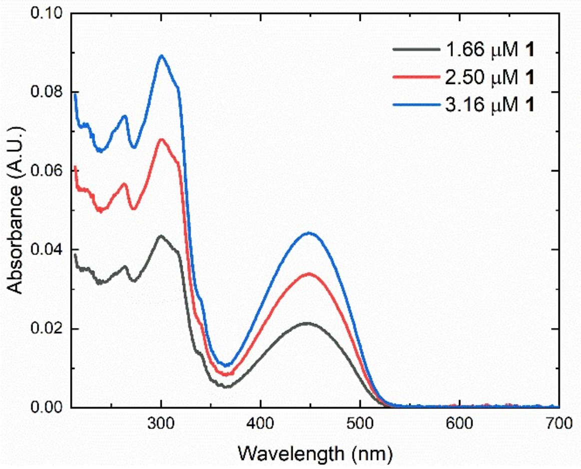

Figure S99. The UV-visible absorbance spectra of compound **1** at varying concentrations, used in the calculation of the relative quantum yield

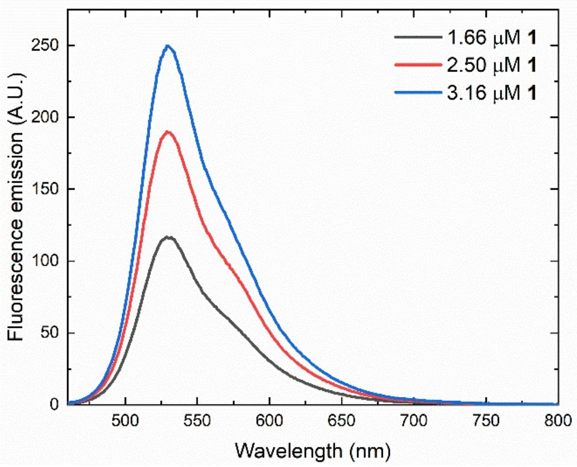

Figure S100. The fluorescence emission spectra of compound **1** at varying concentrations, used in the calculation of the relative quantum yield (λ_{ex} = 450 nm)

Summary Figures for pH Dependent Experiments

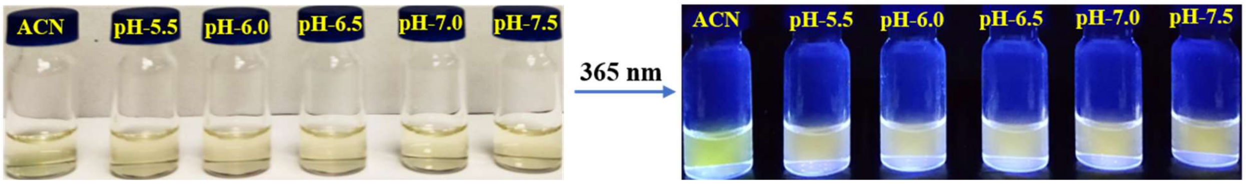

Figure S101. Photographs of solutions of bimane **1** in acetonitrile and under mildly acidic-to-neutral conditions

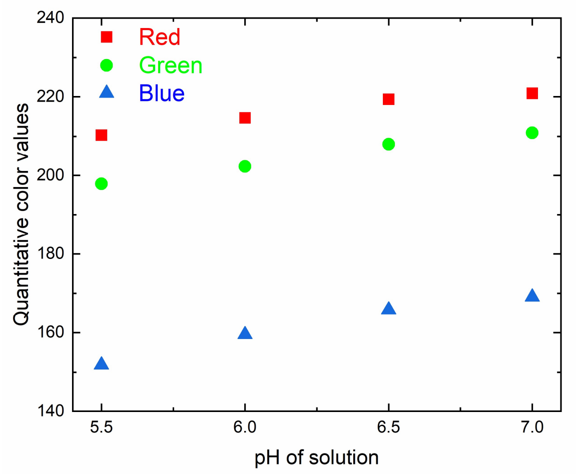

Figure S102. Colorimetric values of the solutions of bimane **1** under ambient light, showing minimal differences in RGB values for mildly acidic solutions

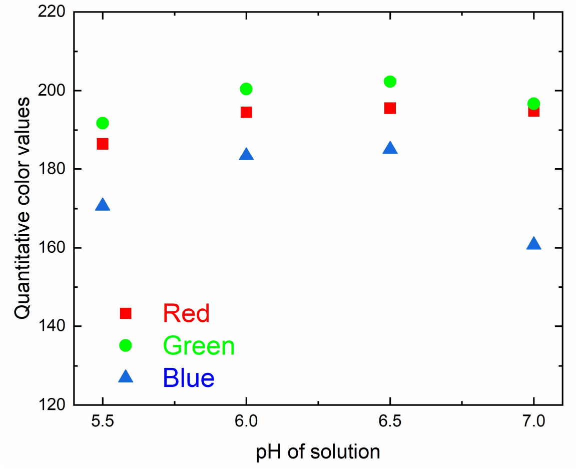

Figure S103. Colorimetric values of the solutions of bimane **1** under 365 nm excitation, showing minimal differences in RGB values for mildly acidic solutions

REFERENCES

[1] Kosower, E. M.; Faust, D.; Ben-Shoshan, M.; Goldberg, I. Bimanes. 14. Synthesis and Properties of 4, 6-Bis(carbalkoxy)-1,5-Diazabicyclo[3.3.0]octa-3,6-diene-2,8-diones [4,6-bis(carbalkoxy)-9,10-dioxa-syn-bimanes]. Preparation of the Parent syn-Bimane, syn-(Hydrogen,hydrogen)bimane. *J. Org. Chem.* **1982**, *47*, 214-221.

[2] Neogi, I.; Das, P. J.; Grynszpan, F. Dihalogen and Solvent-Free Preparation of syn-Bimane. *Synlett* **2018**, *29*, 1043-1046.

[3] Szumski, O.; Karmakar, J.; Grynszpan, F. Re-enter the syn -(Me,I)Bimane: A Gateway to Bimane Derivatives with Extended π-Systems. *Synlett* **2021**, *32*, 1141-1145.

[4] Saute, B.; Premasiri, R.; Ziegler, L.; Narayanan, R. Gold Nanorods as Surface Enhanced Raman Spectroscopy Substrates for Sensitive and Selective Detection of Ultra-Low Levels of Dithiocarbamate Pesticides. *Analyst* **2012**, *137*, 5082-5087.

[5] Lakowicz, J. Principles of Fluorescence Spectroscopy, Third Edition.; Springer: New York USA, **2006**.

[6] Brouwer, A. M.; USE IUPAC Commission Standards for Photoluminescence Quantum Yield Measurements in Solution (IUPAC Technical Report). *Pure Appl. Chem.* **2011**, *83*, 2213-2228.
